# East African cichlid fishes

**DOI:** 10.1186/s13227-022-00205-5

**Published:** 2023-01-05

**Authors:** M. Emília Santos, João F. Lopes, Claudius F. Kratochwil

**Affiliations:** 1 Department of Zoology, University of Cambridge, Cambridge, UK; 2 Institute of Biotechnology, HiLIFE, University of Helsinki, Helsinki, Finland

## Abstract

Cichlid fishes are a very diverse and species-rich family of teleost fishes that inhabit lakes and rivers of India, Africa, and South and Central America. Research has largely focused on East African cichlids of the Rift Lakes Tanganyika, Malawi, and Victoria that constitute the biodiversity hotspots of cichlid fishes. Here, we give an overview of the study system, research questions, and methodologies. Research on cichlid fishes spans many disciplines including ecology, evolution, physiology, genetics, development, and behavioral biology. In this review, we focus on a range of organismal traits, including coloration phenotypes, trophic adaptations, appendages like fins and scales, sensory systems, sex, brains, and behaviors. Moreover, we discuss studies on cichlid phylogenies, plasticity, and general evolutionary patterns, ranging from convergence to speciation rates and the proximate and ultimate mechanisms underlying these processes. From a methodological viewpoint, the last decade has brought great advances in cichlid fish research, particularly through the advent of affordable deep sequencing and advances in genetic manipulations. The ability to integrate across traits and research disciplines, ranging from developmental biology to ecology and evolution, makes cichlid fishes a fascinating research system.

## Natural habitat and life cycle

**Fig. 1:**
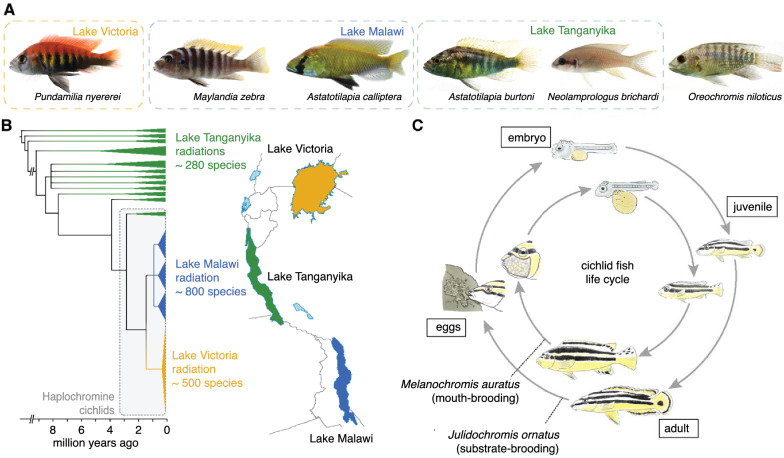
Evolution and Development of East African cichlid fishes. **A** Representatives of East African cichlids for which genomic information is available (Note: the Astatotilapia genus contains multiple paraphyletic species and is therefore found in both Lake Malawi and Lake Tanganyika). **B** Simplified phylogeny of East African cichlids with the radiations of Lakes Tanganyika (green), Malawi (blue), and Victoria (orange). **C** Life cycle of a substrate-brooding cichlid from Lake Tanganyika (*Julidochromis ornatus*) and a mouth-brooding, haplochromine cichlid from Lake Malawi (*Melanochromis auratus*). Photo credits: Ralf Schneider (*A. burtoni* in A)

**Fig. 2:**
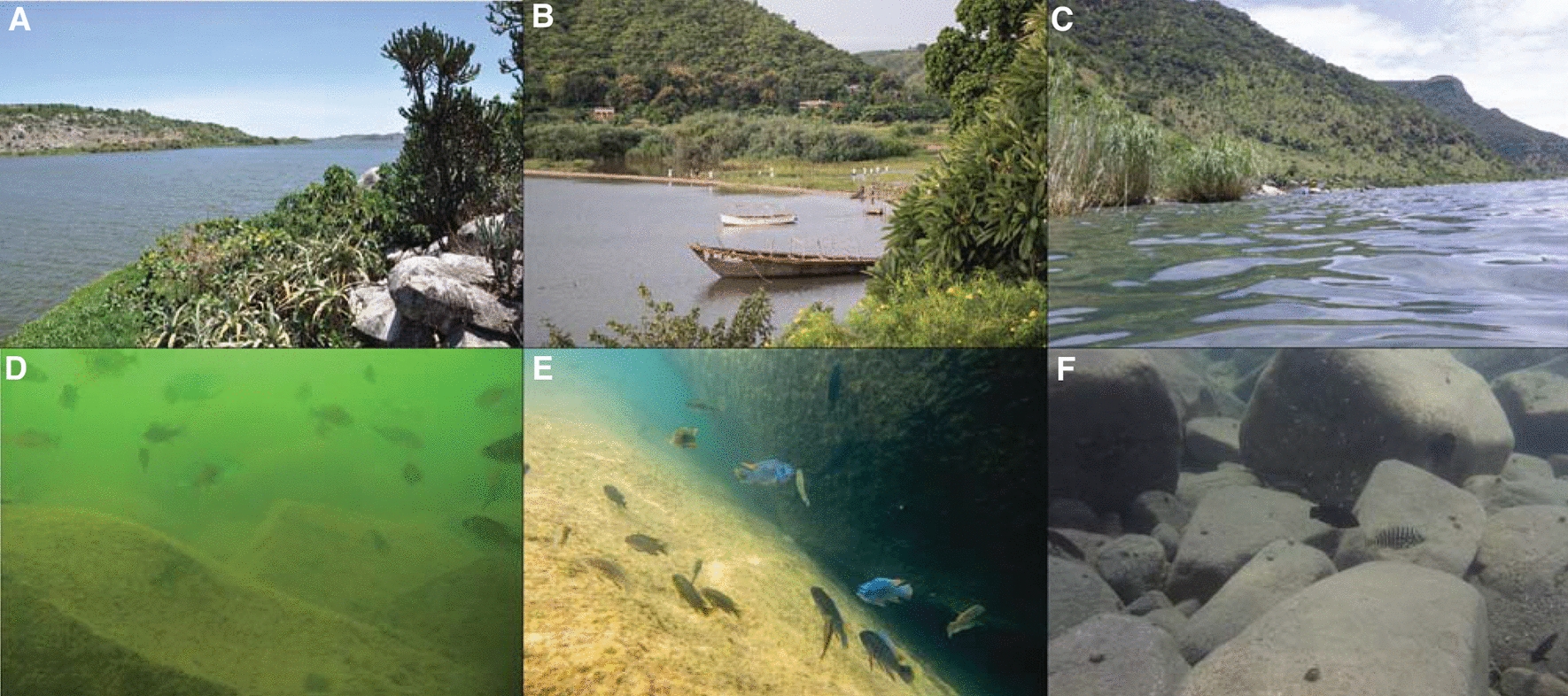
Habitat of East African rift lake cichlid fishes. **A**–**C** Lakes Victoria (**A**), Malawi **(B**), and Tanganyika (**C**) are the hotspots of cichlid fish diversity with over 1200 mostly endemic species. **D**–**F** The waters of the three large rift lakes largely differ in visibility, with Lake Victoria being quite turbid (**D**) and Lake Tanganyika (**F**) and especially Lake Malawi (**E**) being much clearer. Note that the shown habitats are not fully representative of the rich diversity of lake habitats. Photo credits: Joanna Meier and Florian Moser (**A**, **D**), Hannes Svardal (**B**, **E**), Leo Lorber (**C**, **F**)

When diving for the first time in the clear waters of Lake Malawi, one is reminded more of a coral reef than of a freshwater lake, as the lake harbors hundreds of colorful fish species (Figs. 1A and  2E). Lake Malawi is part of a chain of lakes in the East African rift, an active continental rift zone that started to form 20–25 million years ago [1]. The rift gave rise to three of the ten largest freshwater lakes on our planet (Lake Victoria, Lake Malawi, and Lake Tanganyika) (Fig. 1B). The fish fauna within these lakes is dominated by a single fish family, the cichlid fishes (Cichlidae). No other group of fish has been more successful in colonizing these lake environments. In a few million years over 1200 species evolved in the Rift Lakes; most of these species do not exist elsewhere. While Lakes Malawi (Fig. 2B, E) and Tanganyika (Fig. 2C, F) are very clear and deep lakes (average depths 292 and 570 m, respectively), Lake Victoria is relatively shallow (average depth 41 m) and much more turbid (Fig. 2A, D).

In Lake Malawi and Lake Victoria, species of the haplochromine cichlids (Haplochromini), the most species-rich tribe, are predominant. Of the three rift lakes, the Malawi and Victoria radiations of haplochromine cichlids constitute the two youngest, but also most species-rich cichlid radiations (Fig. 1B). In Lake Malawi, the species number is estimated to be between 500 [2] and 860 [3]. These species diverged only within the last 800,000 years [3]. In Lake Victoria, over 500 species evolved in the last 15,000 years [4] after the last desiccation of the lake [5]. Lake Victoria cichlids therefore have one of the highest speciation rates of all vertebrates [4]. In Lake Tanganyika, the oldest of the three lakes, roughly 240 species and 16 tribes have been described [6].

Cichlids exhibit a variety of breeding and parental care behaviors. While almost all cichlid species exhibit rather strong parental care, it is the haplochromine cichlids (and some other tribes including for example some species that belong to the non-haplochromine tilapia fishes) that show a particularly remarkable parenting behavior referred to as mouth-brooding. Courtship behavior in these haplochromine mouth-brooding cichlids is complex and involves a crucial stage where the female picks up the unfertilized eggs into her mouth. Male cichlids have “egg-spots”, yellow-to-red markings on the anal fin that mimic the eggs [7, 8]. During courtship, the female will attempt to take up these “egg dummies'' alongside the real eggs, at which point the male fertilizes the eggs within the female’s mouth [7, 8]. This cycle repeats several times, ensuring high rates of fertilization. Under constant movement the embryos develop in the mouth of the mother, from which they only leave after 2 weeks or more. Relative to substrate breeders, mouth-brooding cichlid larvae tend to have a much larger yolk sac as they develop in the protected environment of the mother’s mouth (Fig. 1C). The non-haplochromine tribes of Lake Tanganyika are mostly substrate breeders. In these species, the eggs are adhesive and attached to stones or within crevices, where they are guarded, cleaned, and fanned by the parents (Fig. 1C). A particularly unusual form of substrate breeding is shell breeding, where eggs are laid in empty shells of gastropods [9]. Consequently, because of this variation in parental care behaviors, the number of eggs per female also differs drastically, from 10 to 80 in mouth-brooding cichlids, to up to hundreds or even more than a thousand eggs in substrate-brooding cichlids [9].

There are several descriptions of different aspects of cichlid fish ontogeny. These include detailed developmental staging guides for the Nile tilapia *Oreochromis niloticus* [10] and the haplochromine cichlid *Astatotilapia burtoni* [11] (Fig. 1A). Furthermore, there are concise developmental descriptions for a few of other species [12–15] and more targeted ontogenetic descriptions focused on early embryogenesis [16], coloration [17–19], fin development [20], skeleton development [21, 22], and gene expression [23]. Development of cichlids is similar to other teleost species, although early development in cichlids is two to three times slower in comparison with zebrafish, as shown for the African non-haplochromine cichlid *Oreochromis niloticus* and the Neotropical Midas cichlid *Amphilophus citrinellus* [24]. One of the most important differences compared to many other teleost species is that cichlids undergo “direct development” when transitioning to the adult form [11], meaning that they do not pass through a free-feeding larval stage (such as in zebrafish) [25]. Consequently, many traits that are not present in intermediate larval forms of indirectly developing species develop relatively early and directly into the adult form in cichlids (e.g., fin structures [11]). After 2 to 3 weeks, the juveniles start to feed. During this period, mouth-brooding cichlids leave the mother’s mouth for increasing periods of time until they completely separate. Cichlids are sexually mature after 4 months (e.g., some *Astatotilapia burtoni* lab strains) to up to a year or longer for larger species. Cichlids undergo indeterminate growth, meaning that they grow rapidly during ontogeny but also continue to grow as adults. Adult East African cichlids are usually between 5 and 25 cm in length (i.e., standard length, measured from snout tip to caudal fin base). Some piscivorous predators, such as *Buccochromis lepturus* in Lake Malawi and *Boulengerochromis microlepis*, can reach up to 40 cm and 90 cm in length, respectively. It should be noted that life history traits such as egg size, clutch size, fecundity, maturation rates and sizes, and care type and duration greatly differ between cichlid species, particularly when substrate- and mouth-brooding species are compared [26–28].

## Field collection and laboratory culture

### Laboratory culture

**Fig. 3:**
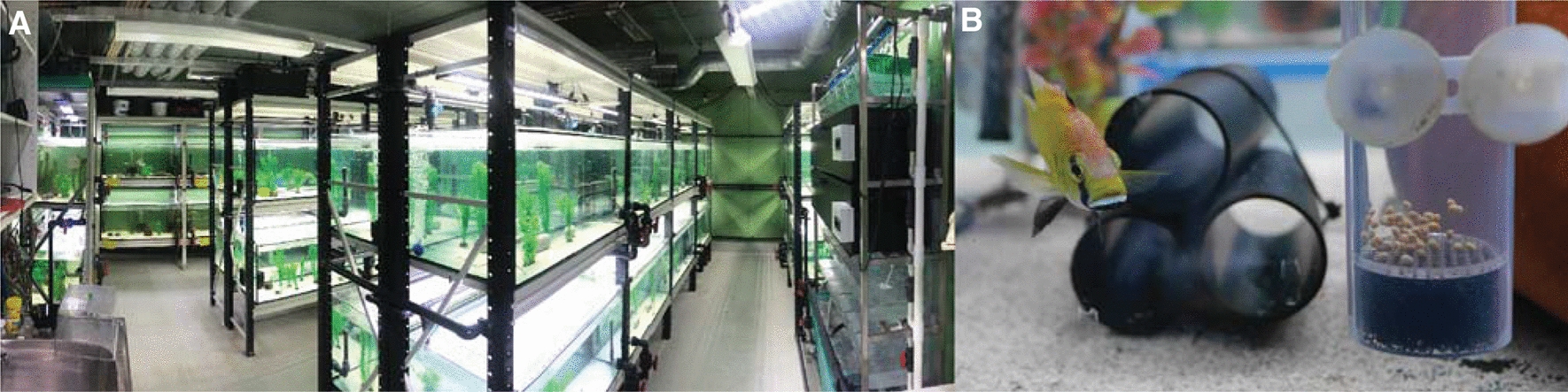
Laboratory culture. **A** Example of a cichlid fish facility with 240-L aquaria and a zebrafish rack for raising juveniles (right) (University of Helsinki). **B** An *Astatotilapia calliptera* male with hiding tubes and an egg tumbler for raising embryos and juveniles (University of Cambridge)

Most East African cichlid species can be reared in an aquarium setting and will breed in captivity (Fig. 3). Cichlids should generally be kept at temperatures between 22 to 28 ℃ under a 12-h dark–light photoperiod, although shorter day cycles are possible (e.g., 16-h dark 8-h light cycle to e.g., reduce algae growth). Lacustrine species thrive in hard, alkaline water, while riverine species prefer softer water. Fish can be fed at least once a day but should be fed at least twice a day if frequent breeding is required. Adults can be fed a mixed diet of flakes, pellets, and bloodworms. The specific nutrient content of dry food will depend on the habitat and natural diet of each species (e.g., a plant-based diet for algivorous and an animal protein-based diet for carnivorous cichlids). A juvenile diet consists of crushed dry food or freshly hatched brine shrimp nauplii.

A variety of aquaria systems can be used, ranging from independent, isolated, or semi-isolated aquaria (Fig. 3A) that require weekly or biweekly water changes, to more sophisticated setups such as circulating systems with a filtration unit or flow-through systems with a constant influx and outflow of fresh water. Tank size depends on the number, age, and size of the fish. Cichlid fry (newly hatched fish) can be reared in small aquaria (approximately 20 L), but must be moved to larger tanks once they reach 1 to 2 cm to avoid stunted growth due to competition for food and space. Adult fish can be kept at higher density breeding groups of 30 to 60 fish in an aquarium of at least 200 L. Cichlid males are known for their territoriality and aggressive displays. Accordingly, to reduce hostile behaviors, breeding stocks should not be kept at excessively low densities. Tank environments should ideally be enriched with artificial or real plants, hiding plastic tubes, and sand substrate. Males can be provided with a clay pot in which they can establish a territory that also serves as a spawning site for gravid females.

If egg collection at the first-cell stage is required, crosses can be set up between one male and 8 to 15 females, with the male and females separated by a transparent perforated divider. The divider can then be removed, and fish interactions monitored for spawning. If spawning is detected, the fish should be given an additional 30 to 90 min (depending on the species) to fertilize the eggs. Healthy adult females generally spawn every 4 weeks, but this may vary between species [29]. Note that this crossing strategy has to our knowledge only been attempted in mouth-brooding cichlids and remains to be tested for substrate brooders. For larger substrate-brooding species, abdominal stripping (removing the eggs by gently massaging the abdomen) is an alternative approach to obtain eggs that can then be fertilized in vitro using sperm obtained by the same procedure in males [30].

Mouth-brooder embryos can be collected at any point in their development, from first-cell stage eggs to free-swimming juvenile stages. To do this, females are collected with a net and hand-held above a small water container. The embryos are removed either by gently massaging and opening their jaws or by gently spraying water in their buccal cavity with a small plastic pipette. Embryos can then be raised in cichlid egg tumblers in an aquarium or moved to 6-well plates (Fig. 3B). The tumblers provide a constant flow of oxygen-rich water that can be regulated to sustain a gentle egg motion that mimics the mouth incubation movements and prevents fungal and bacterial infections. When reared in 6-well plates, the eggs (especially when they have been microinjected or manipulated) should be cultured in aquarium water with antifungal and antibacterial agents, such as methylene blue (10 mg/ml) and penicillin/streptomycin (Sigma P4333 with 10,000 units penicillin and streptomycin 10 mg/mL; diluted 1:1000). Plates should be kept in an orbital shaker at temperatures between 25 to 28 °C. Daily water changes are required. Although less movement and oxygen are required, substrate-brooder embryos can also be kept in tumblers or large petri dishes and 6-well plates on an orbital shaker.

### Field collection

Cichlids are a very popular system in the fields of speciation genomics, ecology, and behavior. Accordingly, field work is an essential part of this research. Field work is often performed in collaboration with local teaching and research institutions (e.g., University of Malawi and Tanzanian Fisheries Research Institute), which have provided excellent local expertise in both species’ distribution and identification. Local permits and a Nagoya protocol should always be in place. Cichlids can be collected in a variety of ways depending on the habitat. Collection can be performed with seine nets in shallow waters, whereas scuba diving is required in deeper waters. Fish are then chased into nets and collected into net bags for further studies (e.g., for phenotyping or fin clip collection for genomic studies). A large proportion of East African cichlid species are philopatric and stay in or regularly return to a particular area. It is thus feasible to conduct behavioral observations, collect genetic material, tag individuals, and conduct release and recapture experiments to follow wild individuals and populations through time [31]. If species are not philopatric, field-based cage experiments that can harbor dozens to hundreds of individuals are still possible [32].

## Major interests and research questions

### A model system for many disciplines and integrative research

Almost as diverse as cichlids themselves are the questions that have been studied in cichlid fishes. Cichlids have become a model system for studying ecology, evolution, genomics, genetics, development, and behavioral biology and for questions that integrate across disciplines. Cichlids are phenotypically highly diverse and can be easily studied in the wild and the lab. Due to the recent divergence time (most haplochromine species diverged in the last few hundred thousand years), species can often be hybridized in the lab, permitting the identification of the genetic basis of adaptive traits. Furthermore, cichlids are also amenable to genetic manipulations and developmental analysis. One further advantage of the cichlid model system is that there is a rich literature on their ecology and their behavior in the wild [33–35], which will only be discussed superficially in this review. The fact that knowledge exists for so many research disciplines, including behavioral, community, and ecosystem ecology allows integration across levels of biological organization, from genes to phenotypes to individuals, populations, and communities.

### Understanding the cichlid phylogeny: from trees to networks

The phylogenetics of East African cichlids have been investigated using molecular markers since the early 1990s [36, 37]. Over this 30-year period, cichlid phylogenetics has moved from species trees based on single mitochondrial genes [36, 37], sets of mitochondrial or nuclear markers (or both) [38–41], marker sets from reduced representation sequencing, such as RAD-seq [42, 43] or hybrid capture-based approaches [44], to fully resequenced genomes [2–4, 6]. The generation of these phylogenies is currently no longer limited by the number of markers but by the complex evolutionary history of cichlid fishes [3, 38, 45, 46]. Insights from reduced-representation and whole-genome sequencing made it evident that cichlid radiations are not tree-like and cannot be understood as a series of branching events [3, 4]. The evolutionary histories of cichlid fish radiations are therefore challenging to reconstruct due to the prevalence of incomplete lineage sorting in combination with introgression and hybridization that occurs frequently in cichlids. In recent years, a major focus has therefore been on the identification of hybridization and introgression events and how they might have influenced the adaptive radiations of cichlid fishes and their speciation and diversification [3, 44–49]. For example, there is ample evidence that the radiations of Lake Victoria [45], Lake Malawi [46], and Lake Tanganyika [44] may have been driven by ancient hybridization events. However, patterns of introgression and hybridization are so complex that they cannot be fully described, especially in young lineages such as in the very young radiation of Lake Victoria cichlids (likely less than 15,000 years [5, 50]). Therefore, these young radiations (evident also for young crater-lake radiations [51, 52]) are now often represented as phylogenetic networks and not trees [4].

### Why are there so many cichlid species?

One of the central questions regarding cichlids and their species-rich adaptive radiations is why there are so many cichlids. Currently, there are 1712 taxonomically valid cichlid species [4], but the real number is likely much higher. In particular, diversification rates of East African Rift Lake cichlids are almost unparalleled [4] and it remains puzzling why so many cichlid species evolved in these lakes while other lineages of teleost fishes or other clades did not radiate similarly. Ecological opportunity (i.e., availability of “evolutionarily accessible resources little used by competing taxa” [53]) is a key factor in explaining the diversification in the lakes. Comparative studies suggest that ecological factors (such as lake depth) and intrinsic factors (such as traits linked to sexual selection) might affect the propensity of speciation [4, 54]. Additionally, several other phenotypic, evolutionary history, and genomic characteristics have been proposed to play a role. These include phenotypic plasticity [55], evolutionary innovations such as the pharyngeal jaws [56], a complex population history that involves ancient hybridization, high degrees of incomplete lineage sorting and frequent introgression events [2, 38, 44–47], and genetic features such as transposons, miRNAs, structural variation, and rapid *cis*-regulatory evolution [2, 4, 57–59]. Furthermore, changes in the visual system of Lake Victoria cichlids (see “*Sensory system biology*” section below) constitute a well-accepted example of sensory drive (i.e., divergence in communication systems driven by environmental variation) as a facilitator of speciation [60]. An important area for future research is to investigate in detail how these genomic features and phenotypic traits influence the dynamics of pre- and post-zygotic isolation and how this might ultimately lead to reproductive isolation and speciation [61].

### General themes: loci of repeated and diversifying adaptive evolution

The parallel adaptive radiations of cichlid fishes also provide the means to analyze general patterns of evolution and to investigate the genomic loci that underlie evolutionary adaptations. An interesting phenomenon is that similar phenotypes have evolved independently in several of the lakes, constituting a case of convergent (or parallel [62]) evolution. These evolutionary replicates offer an opportunity to investigate if they are shaped by similar genetic architectures (monogenic, oligogenic, polygenic) [63, 64], gene networks, or even the same or similar genes and mutations [62, 65–68]. For example, it has been shown that stripe color pattern evolution is driven by the same major-effect gene, *agouti-related peptide 2 (agrp2),* in the different East African cichlid radiations [68]. The genetic underpinnings of these cichlid phenotypes are mainly studied using genome-wide association mapping in populations and qualitative/quantitative trait loci mapping in pedigrees (see “*Experimental approaches**”* section). The relative importance of regulatory and coding evolution can also be studied through these approaches [70, 71]. Lastly and more generally, the molecular mechanisms that underlie adaptive traits are increasingly studied, including miRNAs [2, 72–74], transposons [2, 57], epigenetic modifications [75, 76], and structural variation [77–79]. For example, very recently it has been shown that transcriptional changes of ecologically relevant genes are often driven by transposon insertions that then cause epigenetic modification (i.e., DNA methylation) [75]. We discuss many of the traits investigated below.

### How colors and color patterns evolve

**Fig. 4:**
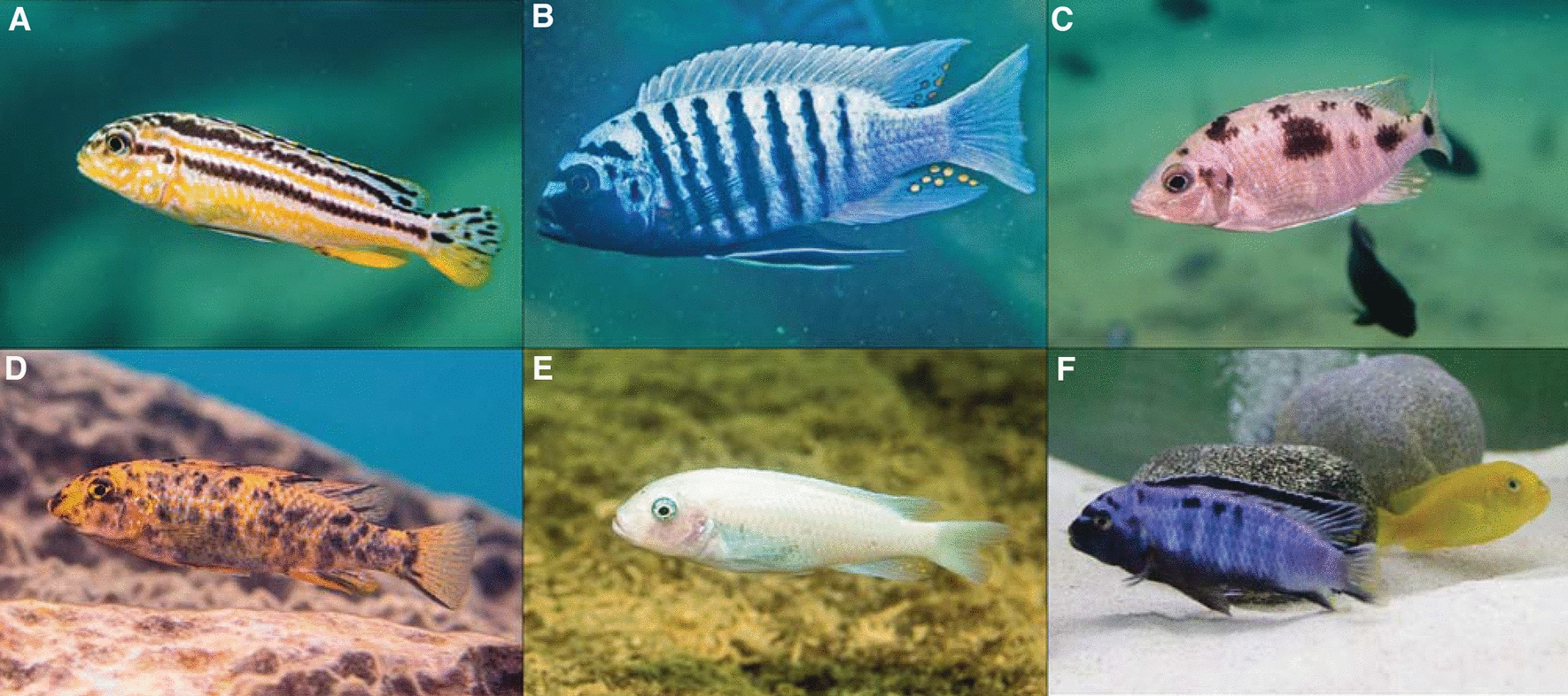
Coloration phenotypes in cichlid fishes. **A** Horizontal stripe patterns in *Melanochromis auratus* (stripes). **B** Vertical bar patterns and egg-spots in *Maylandia zebra.* **C** Spot patterns in *Otopharynx* sp. “heterodon nankhumba”. **D** The orange blotch (OB) phenotype in *Labeotropheus trewavasae.* **E** Amelanism in *Maylandia callainos.* **F** Sexual dimorphism in *Pseudotropheus saulosi* with a blue male and yellow female. Photo credits: Hannes Svardal (**A**–**E**), Muktai Kuwalekar (**F**)

**Fig. 5:**
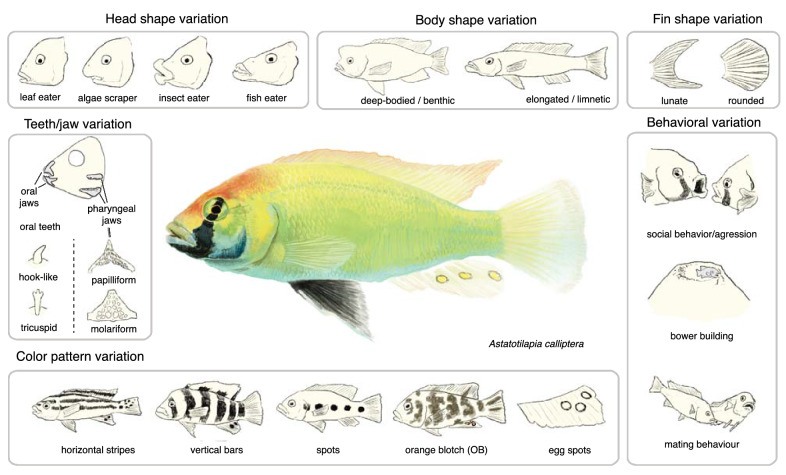
Axes of divergence in cichlid fishes. A selection of phenotypic traits and their variation in cichlid fishes. For example, highly diverse traits include trophic adaptations such as head shape (including the evolution of hypertrophied lips in crevice-feeding insect eaters) and teeth and jaw variation. Moreover, cichlids exhibit great variation in body shapes and fin morphology and variation in color patterns (including egg-spots) and behaviors, such as mating rituals and social behaviors

The diversity of hues and color patterns in cichlid fish is one of their most striking traits (Figs. 4 and 5). Especially in the clear waters of the Rift lakes, sexual selection and also natural selection has shaped an explosive diversification of coloration. Not surprisingly, coloration traits have been a prime target for genotype–phenotype mapping. The traits that have been most intensively studied are melanic patterns such as stripes (horizontal; Fig. 4A) and bars (vertical; Fig. 4B), the haplochromine-specific egg-spots (Fig. 4B), spots on the body (Fig. 4C), the orange blotch polymorphism (Fig. 4D), amelanisms (Fig. 4E), morphological and physiological color change, and the conspicuous nuptial coloration of sexually dimorphic species (Fig. 4F).

Most cichlids have either vertical (almost two thirds of the species) or horizontal (roughly one-third of the species) melanic patterns [80, 81]. The evolution of these two traits is correlated with morphology (body elongation), behavior (shoaling), and habitat preference (vegetation) [80, 81]. Mechanistically, the presence and absence of stripes have been linked to regulatory variation of a single gene, *agouti-related peptide 2 (agrp2),* an antagonist to melanocortin signaling [69, 82]. In Lake Victoria, radiation stripes are a Mendelian trait [69], whereas in the Lake Malawi radiation additional modifier loci have evolved (or been retained), resulting in more variation of stripe patterns (e.g., variation in stripe number and continuity) [83]. Evolutionarily, the *agrp2* locus is highly dynamic [77], with the exact *cis*-regulatory mechanisms of *agrp2* regulation differing between Lakes Malawi (variants in the 5′ untranslated region) and Lake Victoria (intronic regulatory element) [69, 84]. The developmental mechanisms of stripe formation are less understood, but it has been suggested that the migratory pathways of melanophore precursors between myosepta (i.e., the connective tissue separating myomeres, or the blocks of skeletal muscles) prepattern the trait [19]. This prepattern restricts where pigment cells populate the skin, which may constrain the variation in horizontal stripe number to one to three stripes. In contrast, for vertical bar patterns there is no clear understanding about the genetic basis of variation in their presence except that it is likely polygenic [83]. Beyond the absence or presence of bars there is also no knowledge about genetic factors that influence their variation in number, position, width, or other characteristics. Interestingly, compared to stripes, bars are more variable in number and size, sometimes even within species [83]. Their formation may be driven by multiple cellular mechanisms that affect chromatophore density, pigment production, and intracellular pigment dispersal [18, 19]. During ontogeny, bars develop much earlier than stripes [85].

Egg-spots are another trait that have received considerable attention. These yellow-to-red oval ornaments are predominantly found on the anal fin of haplochromine cichlid males. There is a rich literature on the functional role of egg-spots since the early description by Wolfgang Wickler in 1962 [7], with partially conflicting results regarding the selective agents (under sexual selection or not), heritability of egg-spot number and color (plastic or genetically determined), and role in mate choice and male–male competition (important or not important) [17, 86–88]. Several genes have been linked to the evolution of egg-spots, including adaptive coding sequence evolution of the cell-surface protein *colony-stimulating factor 1 receptor a (csf1ra)* [89] and regulatory evolution of *four and a half LIM domains 2 a/b (fhl2a/fhl2b)* [8, 90]. Interestingly, regulatory evolution of *fhl2b* has been linked to a transposon insertion in the promoter region of the gene that leads to a gain of gene expression in iridophores, a silvery reflective pigment cell type that contributes to egg-spot formation [8].

Another very prominent coloration trait in Lake Malawi and Victoria cichlids is the orange blotch (OB) phenotype. This phenotype is particularly common in females and is defined by irregular melanic blotches (Fig. 4D). One explanation for the sex linkage of the phenotype is that it evolved by sexually antagonistic selection. Females benefit from the more cryptic coloration, while males with the OB pattern would have a disadvantage over males with conspicuous nuptial coloration. It has been suggested that this sexual conflict (i.e., sexes having a different fitness strategy) has been resolved by the evolution of a novel female sex-determining region in close genomic proximity of the OB locus [91]. This leads to a linkage between female sex and the cryptic OB coloration. The orange blotch phenotype is a Mendelian trait [92] that has been linked to an allelic series that is likely causal for the regulatory variation of the gene *paired box 7a (pax7a)* and the aberrant migration of melanophores [91, 93].

The nuptial body coloration of cichlids has also been investigated using genetic mapping approaches [94, 95] and gene-expression analyses [96, 97]. Other coloration phenotypes that have been studied are morphological (slow) and physiological (fast) color changes [98–101]. Here, much remains to be studied with respect to their molecular underpinnings. Comparing both morphological and physiological color change evolution in cichlids will elucidate whether the genetic basis (e.g., coding or regulatory mutations) underlying variation in morphology and physiology might differ.

### Trophic adaptations: jaws, teeth, and head shape

Cichlids harbor substantial diversity in their craniofacial and dental morphology, such as variation in head shape and teeth morphology and number (Fig. 5). This diversity is largely driven by adaptation to distinct trophic environments [102–104]. The trophic apparatus of cichlids consists of two sets of jaws. The oral jaws are responsible for food manipulation and ingestion and the pharyngeal jaws in the throat are responsible for food processing. Cichlid pharyngeal jaws (Fig. 5) exhibit several morphological properties that facilitate their processing efficiency and adaptability. Accordingly, it is believed that these acted as a “key innovation'' that allowed cichlids to invade and explosively diversify in a variety of trophic niches [56]. Despite the importance of pharyngeal jaws for cichlid evolution, most work has focused on mapping differences in oral jaw morphology, which is strongly associated with head shape. For example, quantitative trait loci mapping (QTL) of variable oral jaw morphologies between Malawi cichlid species identified *patched 1 (ptch1), bone morphogenetic protein 4 (bmp4)*, and *limb bud-heart (lbh)* as candidate genes associated with shape variation [105–107]. More recently, it has also been shown that the oral and pharyngeal jaws are evolutionarily coupled. This integration was mapped to a pleiotropic locus, *mothers against decapentaplegic homolog 7 (smad7)*, which is proposed to shape both sets of jaws [108]. The coupling of jaws is thought to have contributed to the evolutionary success of cichlids by facilitating rapid and concerted shifts when adapting to different foraging habitats [103, 108]. Variation in jaw morphology is paralleled by variation in the number and shape of teeth. Several candidate genes (e.g., *secreted frizzled-related protein 5 [sfrp5]* and *bone morphogenetic protein binding endothelial regulator [bmper]*) were identified to contribute to oral dentition variation [109]. Furthermore, the same genomic regions are associated with both variation in oral and pharyngeal dentition number [110, 111]. These results provide further evidence for the still-debated hypothesis that jaw integration rather than independence may have been a key to the rapid trophic adaptation of cichlids [110, 111].

Notably, craniofacial morphologies (but also variation in pigmentation; see previous section) have a strong developmental link to a single population of cells, the neural crest. These cells emerge from the vertebrate dorsal neural tube early during development, delaminate, and undergo some of the longest migrations of any embryonic cell type to give rise to multiple derivatives such as pigment cells, neurons, and glia of the peripheral nervous system, smooth muscles, craniofacial cartilages, and bones. Differences in neural crest cell migration are associated with variation in cichlid jaw morphologies [105]. This suggests that variation in early neural crest development can contribute to species-specific differences and make cichlids an interesting alternative model system for biomedical research to, for example study basic cellular processes leading to morphological variation [102].

Lastly, another still mostly unexplored trait that is highly correlated with adaptation to distinct trophic environments is body shape (Fig. 5) [112]. The differentiation between limnetic and benthic forms is a common theme throughout cichlid divergence and is associated with the early stages of cichlid radiations [113, 114]. A recent effort to map such variation used two different inter-specific crosses along the benthic–pelagic ecomorphological axis. In total, 55 loci contributing to variation of this trait were identified in these two crosses [115]. Surprisingly, there was no overlap between the candidate genes from both crosses, suggesting that the genetic basis of body shape is highly polygenic and differs between species.

### Fins and scales: upcoming model traits for evo-devo research

Many other traits show extensive inter-specific variation and have been the focus of recent investigations. Fin shapes (Fig. 5) vary greatly across teleost fishes and within cichlids. Furthermore, due to their direct development [11], cichlid fishes are a suitable model to investigate the mechanisms of fin development. For example, a recent study investigated the genetic networks that shape the evolution and individualization of spiny and soft fin rays [20]. Other studies have also described the transcriptional and developmental changes that drive fin shape development and evolution (Fig. 5) [116–118]. For example, many genes, including growth factor and WNT pathway genes, are differentially expressed across fin regions [114, 115]. Moreover, *wnt7aa* and *alpha-1 type I collagen* (*col1a1*) are linked to pectoral fin ray number variation [116]. More research is needed to understand how these genes are causing phenotypic changes mechanistically. Another trait that has been studied from a gene regulatory and evolutionary perspective are scales, which also show extensive variation between species [119, 120]. For example, *fibroblast growth factor receptor 1b* (*fgfr1b*) has been linked to scale shape variation [119]. Studying such traits will elucidate the genetic and developmental mechanisms underlying the astonishing variation present in both fin and scale shape in teleost fish.

### Sensory system biology

Foraging and mating behaviors rely on multiple sensory systems, including vision, olfaction, hearing, and mechanosensation [121]. The most well-studied cichlid sensory system is the visual system. Color vision is critical for reproductive success and is essential for adaptation to environments with varying light regimes. As such, the visual systems of cichlids are highly diverse and have evolved specific visual sensitivities that match their ecology and habitat [122]. Variation can arise from differences in cornea and lens transmission, differences in number and distribution of photoreceptors, and expression and sequence variation of light-sensitive opsin proteins. All these changes can affect visual perception. For example, close relationships between opsin sequence changes and species light environment have been documented, such as spectral shifts towards blue light in opsin genes of Lake Malawi and Lake Tanganyika deep-water species [123, 124]. Furthermore, several opsin protein sequences operate under positive selection. Multiple *cis*-regulatory sequences also show signals of divergence [57, 125, 126]. Importantly, visual adaptation to variable light environments affects female mating preference and speciation patterns. For example, long-wavelength shifts in the murky waters of Lake Victoria led to changes in mate preference and thereby led to sympatric speciation of “red” shallow (*Pundamilia nyererei*) and “blue” deeper water species (*Pundamilia pundamilia*) [60] (i.e., speciation by sensory drive).

Other less studied sensory systems include olfaction, hearing, and the lateral line. Cichlid olfaction is involved in recognition of kin [127] and conspecifics [128–130] and in the identification of female reproductive status and male social rank [131–133]. Further, olfaction may be involved in imprinting, presumably influencing mate preferences [134, 135]. The olfactory organ, which contains the olfactory sensory neurons, is located in the nasal cavity. These neurons are directly exposed to the aquatic environment and contain several transmembrane olfactory receptors. Odorant receptors (ORs) and vomeronasal receptors type 1 (V1Rs) and type 2 (V2Rs) bind to odorant molecules and elicit a response to the odor cue [136]. Cichlids have a large and variable number of such receptor proteins. V1Rs genes show evidence of positive selection, which may suggest an important ecological and behavioral function in cichlid adaptation and speciation [137–140].

Cichlids also communicate through acoustic signals and produce a variety of sounds that are associated with agonistic interactions [141, 142], courtship behavior, mate preference [143], and maintenance of species barriers [144, 145]. Variation in sound detection can result from morphological differences in their inner ear (direct stimulation) or in their swim bladder (indirect stimulation). The swim bladder contains gas that is less dense than that in the fish body. When in contact with sound, this gas vibrates, transmitting the energy to the inner ear [146]. In many teleost fishes, there are modifications of the bladder or cranial morphology that increase this indirect ear stimulation and increase sound detection [147]. Variation in both the inner ear and swim bladder morphology has not been studied extensively in East African cichlids and hence the scope of its diversity remains unknown [148].

Finally, the lateral line is a mechanosensory system that senses hydrodynamic stimuli in aquatic habitats [149]. It provides information about current flows, presence of obstacles and detection of conspecifics (e.g., shoaling and schooling), and presence and identity of prey and predators. In fishes, the lateral line comprises two receptor classes, the canal neuromasts and the superficial neuromasts, which detect differences in water movements and pressure. The superficial neuromasts are distributed throughout the surface of the head, trunk, and tail and are thought to mostly assess the direction and speed of water currents. In comparison, the canal neuromasts are located in pores in the bones of the head and are thought to detect high-frequency pulse changes in water movement, such as prey or predator presence [150, 151]. Both canal and superficial lateral line components vary in morphology across species and are associated with variation in craniofacial morphology (e.g., shape of oral jaws) and dietary habitats [152]. Species with enlarged pores show a higher sensitivity to water flow, which increases their ability to detect prey in the dark and below the surface [153]. Taken together, the association between lateral line morphology and dietary behaviors suggests that divergence in lateral-line systems contributes to the evolution of different feeding strategies. The loci underlying cichlid lateral line variation remain unidentified, but comparisons of canal neuromast development between species with wide and narrow pores suggests that heterochronic shifts in canal growth and morphogenesis contribute to adult trait differences [154, 155].

### The evolution of sex

Fishes are well known for their rich diversity in sex-determination systems, and cichlids are no exception. Many sex-determination systems have been found in cichlids, including different sex loci on different chromosomes, male and female heterogametic systems (XY and ZW), and both monogenic and polygenic sex determination [156]. In fact, cichlids have the highest rates of sex chromosome turnover and heterogametic transitions described to date [157, 158]. Multiple sex-determination systems have also been described within single species [93, 159, 160]. For example, three distinct XY loci were recently characterized in interbreeding populations of *A. calliptera* [161].

A better understanding of sex-determining mechanisms provides an important context for understanding the evolution of sexual dimorphism, the evolution of sexual conflicts, and speciation more generally. In a Malawi cichlid with a polygenic sex determination system (*Metriaclima mbenjii*), the different heterogametic combinations (ZZXX females, ZWXX females, ZWXY females, and ZZXY males) result in modular morphological and behavioral polymorphic variation, which is generated by an interplay of sex-linked (e.g., genes linked to the sex-determining gene) and sex-limited mechanisms (e.g., sex-specific hormones) [162]. Finally, the evolution of a novel sex-determining system has also been associated with the resolution of sexual conflict (see paragraph about the OB phenotype in the subchapter “*How colors and color patterns evolve**”*).

### The complex behaviors and brain evolution of cichlid fishes

Cichlids have a very diverse behavioral repertoire (Fig. 5), and it has been argued that cichlid brains are among the most complex teleost brains, with extraordinary cognitive and social learning abilities [163, 164]. Social structures in cichlids can be highly complex and include changes in social hierarchies, whereby status can switch between dominant, subdominant, and submissive [165–167]. The social structure and behavior of cichlids has been particularly studied in *Astatotilapia burtoni*, a haplochromine cichlid from Lake Tanganyika and adjacent rivers (Fig. 1A) [164, 165], and in several species of Lamprologine cichlids from Lake Tanganyika [31, 168]. One particularly interesting behavior is the bower building of some Lake Malawi cichlid species, in which males build little pits or castles to attract females. These behaviors have a genetic basis [168], with initial investigations using population and QTL mapping approaches that revealed a polygenic and predominantly cis-regulatory genetic basis that includes many genes linked to neurodevelopment and neural plasticity [169]. In recent years, there have also been some more in-depth investigations of the neural and genetic basis of the other social behaviors of cichlids, such as courtship behavior, including studies that used genome editing [170, 171]. For example, the androgen receptor copies ARα and ARβ play complementary roles in regulating social status, with ARα controlling coloration and growth and ARβ controlling reproductive and aggressive behavior [171]. Moreover, prostaglandin F2α relays fertility status and orchestrates sexual behavior of females [170]. From an Evo-Devo perspective, cognitive evolution and large differences in brain morphology are linked to differences in brain patterning during development [172, 173]. For example, comparative investigations of gene expression suggest that brain differences may directly link to variation in the expression in brain-patterning genes, such as *six3*, *fezf2*, *shh*, *irx1b*, and *wnt1* [172].

### Interactions with and adaptations to changing environments

One of the most remarkable characteristics of cichlid fishes is their ability to adapt to extreme and changing environments. Many phenotypes in cichlids, including trophic adaptations and coloration, are highly plastic (i.e., a single genotype can generate more than one phenotype) [174]. For example, dentition [175], especially on the pharyngeal jaws of cichlids, is highly plastic and alternate phenotypes can be induced by different diets [176]. Color patterns, such as the eyebars, are modulated by neuronal and hormonal input and can fade or enhance in their contrast depending, for example, on the individual’s position in the social hierarchy [167]. It is still unclear what role plasticity has played in cichlid evolution (i.e., if it has facilitated or hindered speciation as debated in other systems [177]). Another topic for further study that is important for a more comprehensive understanding of the interaction of fish species with their environment is the microbiome. Several studies in recent years have provided insights into this interaction [178, 179]. Lastly, it has to be noted that despite the evolutionary success of cichlids and their ability to adapt to changing environments, many cichlid species are threatened by or are already extinct due to eutrophication [180] or introduction of predatory fish, such as the Nile perch (*Lates niloticus*) [181].

### Out of Africa—more cichlid model systems

Although cichlids of the great lakes of the East African rift are the hotspot of cichlid diversity, Neotropical cichlids from South and Central America, other African lineages, and the outgroups from Madagascar and India have also been studied extensively. Even though they are not the focus of this review, we still would like to mention some of these important systems briefly. Crater-lake systems from East, West, and Central Africa (Lakes Masoko [51], Barombi Mbo [182, 183], Bermin [182], and Ejagham [182, 184]) and from Central America (Midas cichlids from Nicaragua [52, 185]) provide exciting opportunities to study the early stages of speciation. What makes crater lake adaptive radiations more suitable to study early speciation than the large rift lakes is that crater lakes are isolated, have been seeded through only one or few colonization events, that often occurred very recently, and have a more manageable number of species. As such, these systems are suitable for an in-depth analysis of the genomics of speciation. South American cichlids are also highly diverse and offer interesting traits and characteristics for investigation. For example, the genus Apistogramma is a rare case of pH- and temperature-dependent sex determination in teleost fishes [186]. Finally, many studies have addressed the evolutionary history across all cichlids. For example, it was recently found that the diversification of cichlids into Neotropical and African cichlid lineages occurred after the Gondwanan continental split [187].

## Experimental approaches

A multitude of methods for developmental, genetic and genomic, and phenotype and behavioral analyses have been established within recent decades. Here, we will mainly focus on two types of approaches, genotype–phenotype mapping and genetic manipulations, that make cichlids a particularly attractive system for evo-devo researchers. Although they do not differ considerably from other teleost model systems, we also provide a concise overview of developmental and phenotyping tools.

### Genotype–phenotype mapping

**Fig. 6:**
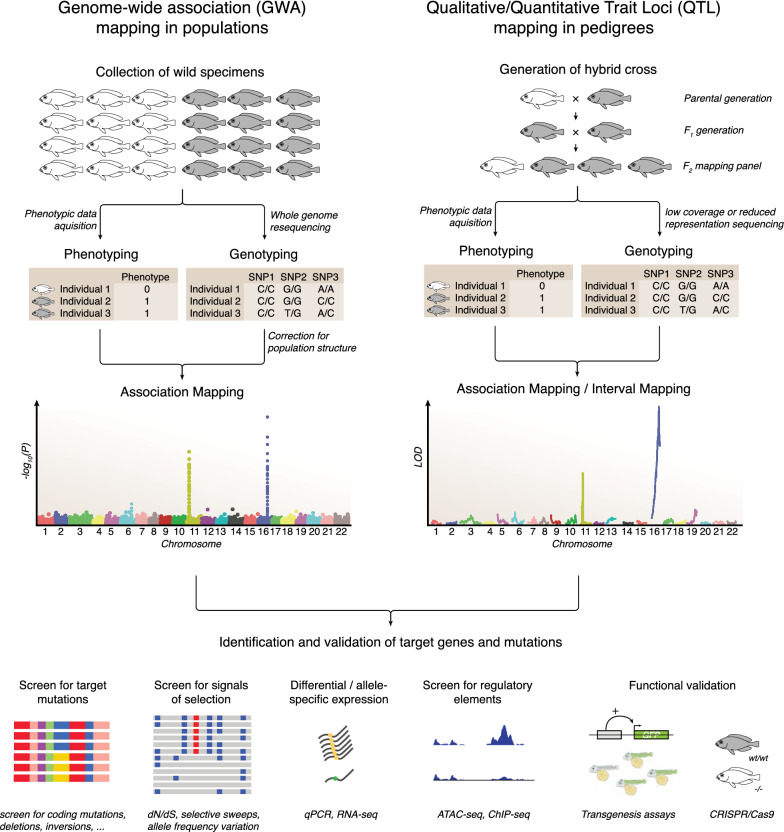
From cichlid phenotypes to genotypes to functional validation. Over the last decade, cichlid fishes have become a prime model to study genotype–phenotype relationships. The ability to collect samples from the field and to conduct hybrid crosses (even between species) makes it possible to identify the genetic bases of traits using genome-wide association (GWA) mapping and qualitative/quantitative trait loci (QTL) mapping, respectively. In combination with other methodologies (especially approaches that include functional validation) candidate genes and mutations can be identified and functionally validated as genes underlying phenotypic variation. Note that for simplicity a simple (qualitative) trait was used in this figure; both analyses can be and are also performed with complex (quantitative) traits

One of the reasons why the cichlid model is attractive for identifying the genetic basis of trait diversity is that the model is amenable to both pedigree-based (qualitative or quantitative trait loci, QTL, reviewed in [188]) and population-based (genome-wide association [GWA], reviewed in [189]) genotype–phenotype mapping (Fig. 6). Both methods have different strengths and weaknesses that we discuss at the end of this section.

For QTL mapping, phenotypically variable individuals from the same or different species are interbred. These first-generation (F_1_) individuals are then interbred again (or alternatively bred with the parents; backcross). The F_2_ generation can be then used to identify genomic loci that associate with heritable traits that vary in the two parents. The results of QTL mapping studies are usually visualized with LOD plots, which show a logarithmic plot of the statistical support (odds ratio) for the association between the genetic markers and the phenotype (Fig. 6). Markers have traditionally been sequenced using reduced representation sequencing approaches (e.g., RAD-seq). An alternative approach that has been used more recently is low-coverage genome resequencing. What makes cichlids such a powerful system for QTL mapping (in contrast to other vertebrate systems) is that many species (especially the young haplochromine species) can still be hybridized and even produce fertile F_1_ hybrids, allowing the unbiased identification of genes that contribute to species-specific phenotypes. Phenotypes that have been mapped in cichlid fishes include craniofacial variation [105–108], color variation [69, 83, 92, 94, 95], fin shapes [118], sex [158], and even behaviors such as bower building [169] (see discussion of the respective traits above).

GWA mapping takes advantage of natural recombination events present between interbreeding populations, or within populations, to map natural variation in traits. As many cichlid species diverged very recently and gene flow often persists to some extent, GWA can be also conducted across species (with some caveats due to population structure, see below). For GWA hundreds or thousands of wild-caught individuals are phenotyped (for the trait of interest) and genotyped (in cichlid fishes usually via genome resequencing). The resequenced genomes are mapped to a reference genome and variants are called. As with the QTL mapping, the combined genotyping and phenotyping data set is used to find associations between genotypic and phenotypic variation. Different methods (e.g., by incorporating a kinship matrix as implemented in EMMAX [190]) can be used to control for population structure, as this can otherwise have confounding effects on the results. This is especially advised if the phenotypes cluster by populations or species (see e.g., [52]). GWA mapping is mostly visualized as Manhattan plots, which show a logarithmic plot of the statistical support (negative logarithm of *P*-value) for each variant (Fig. 6). For now, in African cichlids, only sex determination has been investigated using GWA mapping [161, 191]. However, because of the availability of genomes of hundreds of individuals [3, 4], many studies on other traits will likely be conducted in the coming years.

Both methods, QTL and GWA have advantages and limitations. QTL mapping is limited to the genetic variation that is present in the parents of the cross. Small effect loci mostly remain undetected due to the relatively small number of individuals (approximately 150–500 individuals) obtained in the F_2_ generation. On the other hand, when compared with GWA, the QTL approach suffers less from the risk of false positives. With respect to resolution, QTL mapping is limited by recombination, usually restricting the confinement of target regions to a few hundred kb to a few Mb. For major-effect loci, the size of target regions can be reduced through fine-mapping. The final size of the target region is defined by the recombination breakpoints in these individuals. A further fine-mapping beyond this level of resolution is not possible with this methodology. However, population data (e.g., inter-specific F_ST_ divergence scans using genome resequencing data or targeted sequencing) have been used to further characterize the intervals and to identify causal regions and variants [69, 84, 108]. Regions can be screened for genes with differential gene expression (between species, genotypes in the F_2_, or by screening for allele-specific expression in F_1_ individuals) or signals of selection in wild populations. Lastly, it should be noted that QTL mapping when performed in the laboratory may not target phenotypic variation that is only expressed in the wild (through genotype-by-environment interactions).

In contrast, GWA mapping recovers all genetic variation that can be genotyped based on the reference genome. It can therefore (depending on the sample size) detect both small and large effect loci (at least when using sufficient sample size). False positives can occur due to confounding effects if allele and phenotype frequencies strongly differ between populations. It is possible to partially correct for these effects, but strong population structures can pose challenges. The resolution of GWA mapping is much higher and is only limited by the size of linkage disequilibrium (LD) blocks in the population. It is possible to identify causal variants with GWAS, although often only haplotypes that include multiple target variants are identified. Another caveat of GWA mapping is reference biases that occur if the reference genome does not cover the region where potential causal variants are located and cannot therefore be genotyped. In this case, only variants in close genomic proximity are mapped (i.e., in LD) that would however also show high association if the causal variant is missing due to reference bias [192]. The combination of QTL and GWA mapping can offer a complementary strategy that combines the strengths of both methodologies [52].

In recent years, these genotype–phenotype mapping approaches have been successfully applied and causal regions and genes were identified using additional downstream analyses (Fig. 6 and following sections) to identify and validate the putatively causal genes and mutations (Table 1).

**Table 1:**
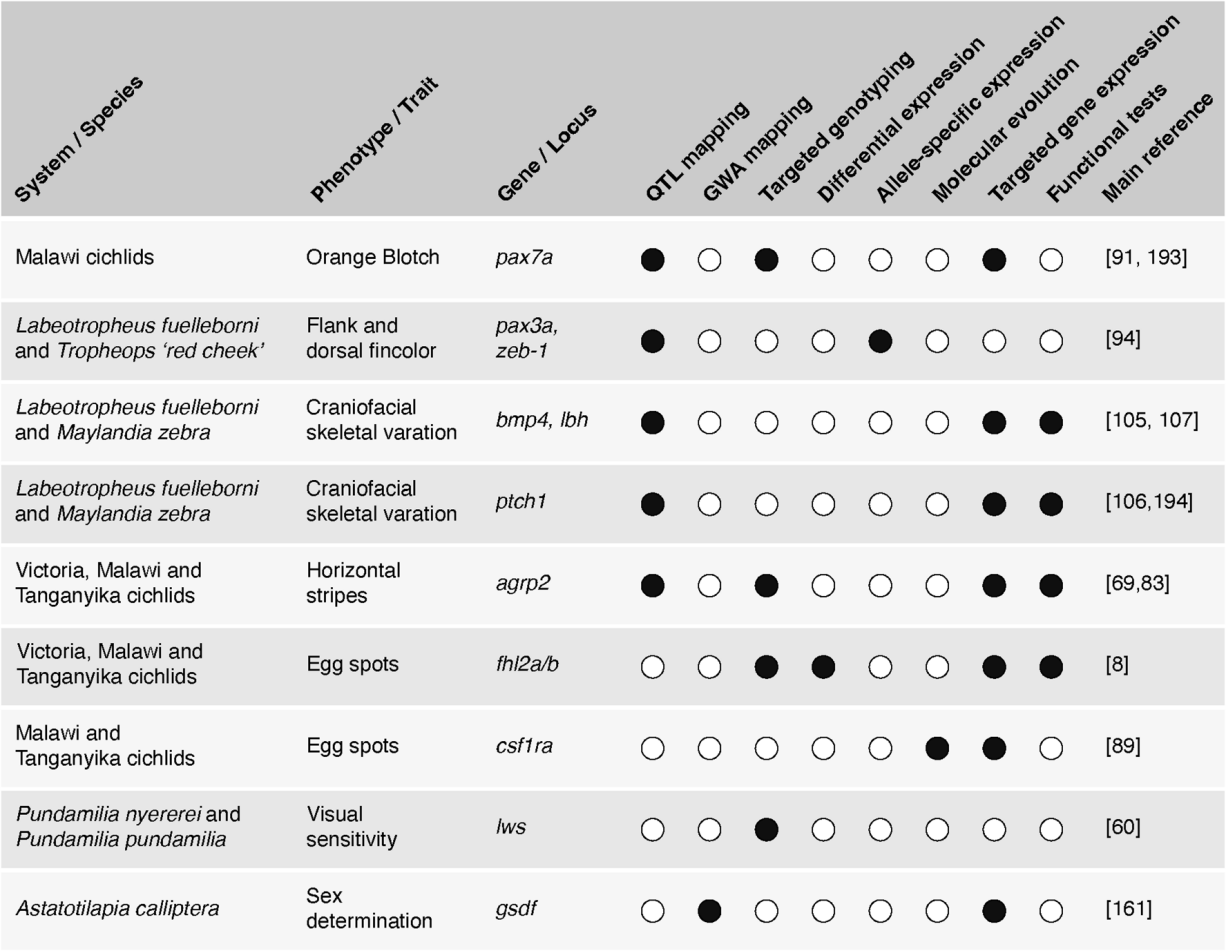
Examples of genotype–phenotype mapping studies in cichlid fishes

We included forward genetic studies that show reasonable support for a causal genotype–phenotype relationship (e.g., use independent experimental approaches, functional validation, or those phenotypes such as opsins with a very direct genotype–phenotype relationship). Targeted genotyping includes Sanger sequencing or microsatellite studies. Gene expression includes qPCR and in situ hybridization. Functional tests include CRISPR–Cas9, transgenesis, and pharmacological manipulation. *QTL* qualitative/quantitative trait loci mapping, *GWA* genome-wide association mapping

### Gene function manipulation

**Fig. 7:**
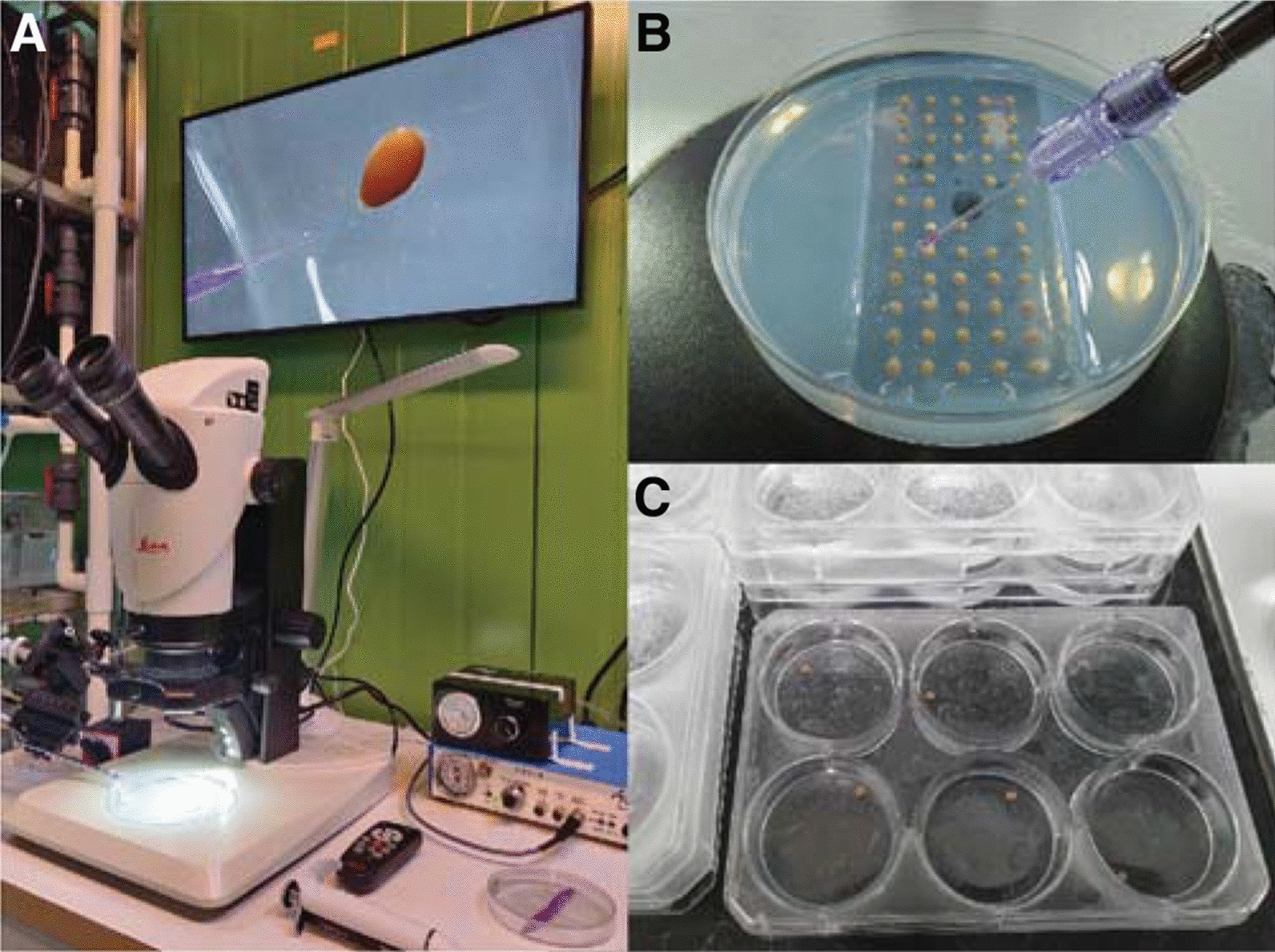
Genetic manipulation techniques. **A**–**C** Over the last two decades, techniques such as Tol2 transgenesis and CRISPR–Cas9 genome editing have been successfully adapted in cichlid fishes. A challenge compared to traditional model teleost fishes (such as zebrafish and medaka) is the small number of eggs per clutch (usually 15–50 eggs), the oval egg shape, and the difficulty in timing fertilization. As in zebrafish, eggs are microinjected using an air pressure-driven microinjector (**A**). Eggs can be held with forceps or put into a supporting agarose mold (**B**). After microinjection, eggs are kept individually in well plates until larvae are free swimming (**C**). Photo credits: Bettina Fischer (**B**)

**Fig. 8:**
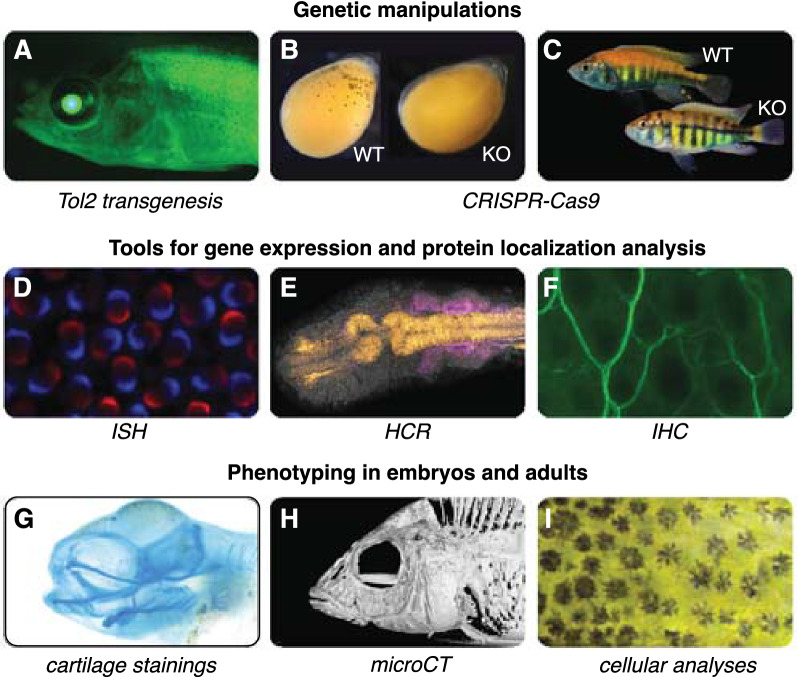
Experimental and phenotyping approaches in cichlids. (**A**–**I**) A wide variety of methodological approaches, including methods available in cichlid fishes that are comparable to other teleost fish model systems. These include methods for genetic manipulations (**A**–**C**), gene expression and protein localization (**D**–**F**), and phenotyping in embryos and adults **G**, **H**. **A** Transgenic cichlid fish of the species *Astatotilapia burtoni* constitutively expressing *GFP* under the *elongation factor 1 alpha, ef1a* promotor. **B** Stable CRISPR–Cas9 knockout of the pigmentation gene *oculocutaneous albinism II, oca2* in *Astatotilapia calliptera* leading to loss of melanin in melanophores. **C** Transient CRISPR–Cas9 knockout of the “stripe gene” *agrp2* in *Pundamilia nyererei,* resulting in the appearance of horizontal stripe patterns in this usually non-striped species. **D** Fluorescent in situ hybridization (ISH) for *rhobdopsin 2b*, *rh2b* and *longwave-sensitive (lws) opsin* in the Malawi cichlid *Maylandia zebra*. **E** In situ DNA-hybridization chain reaction (HCR) for *pax7* (orange) and *SRY-box transcription factor 10, sox10* (magenta) in *Rhamphochromis sp. ‘*chilingali’. **F** Immunohistochemistry (IHC) for nerve fibers on scales of *Melanochromis auratus* using an acetylated tubulin antibody. **G** Cartilage staining of an embryo of *Tropheops sp.* ‘mauve’. **H** MicroCT 3D visualization of *Aulonocara stuartgranti*. **I** Microscopic analysis of melanophore development and patterning in an embryo of the Lake Victoria basin cichlid *Haplochromis latifasciatus*. Photo credits: Scott Juntti (**A**), Joel Elkin / Bethan Clark (**B**), Brian Dalton / Karen Carleton (**D**), Aleksandra Marconi (**E**, **G**), Duncan Edgley (**H**), Jan Gerwin (**I**)

Progress towards understanding the mechanistic basis of organismal diversification has often been hindered by the lack of tools to analyze the functional effects of genetic variation. During the past decade, cichlids have emerged as an integrative model system capable of bridging several levels of biological organization, from variation in DNA sequences to cellular and developmental mechanisms underlying trait variation. Gene function in cichlids has traditionally been studied using pharmacological manipulations using small molecules, such as agonists or antagonists of the target gene or gene pathways [106, 173, 195, 196]. More recently, CRISPR/Cas9 has been used and shown to work at high efficiency in several cichlid species (*Astatotilapia burtoni*, *Astatotilapia calliptera*, *Pundamilia nyererei*, *Oreochromis niloticus*) to target both coding and non-coding sequences [29, 69, 197–199]. DNA, RNA, proteins, or combinations thereof are injected at the single- or two-cell stage into oocytes using microinjection setups (Fig. 7). The targeted species belong to the Malawi (*A. calliptera*; Fig. 8B), Victoria (*P. nyererei*; Fig. 8C), and Tanganyika (*A. burtoni*) radiations and include a riverine outgroup (*O. niloticus*). Some of these studies provided strong support for target genes that control specific traits. For example, knockout of *agrp2* in *P. nyererei* revealed that the gene is responsible for repressing stripes in this normally unstriped species [69]. Moreover, knockout of *ptgfr* demonstrated the importance of the gene in initiating courtship behavior in *A. burtoni* [170].

The next steps are to expand these tool sets to more cichlid species, and more importantly to optimize allelic-exchange protocols to swap single alleles or haplotypes from one species to the other. Finally, the generation of reporter construct lines to test when and where certain non-coding regulatory regions drive gene expression has also been established in *A. burtoni* and *O. niloticus* using the Tol2 transgenesis method (Fig. 8A) [200, 201]. The fact that cichlid clutch sizes are smaller and generation times longer than other teleost systems make both transient analyses and establishing stable lines more challenging. Thus, the development of novel methodologies, perhaps using CRISPR/Cas9-mediated knockins [202], to generate stable reporter lines would be beneficial. It is likely that this will greatly improve in the next few years. The methods can be considered as established for both substrate- and mouth-brooding species, and there are now excellent resources and protocols for microinjections and genome editing in cichlid fishes (see [29] https://cichlidengineering.weebly.com/transgenics.html; last accessed 17 June 2022 for mouth-brooding cichlids and [30] for substrate-breeding cichlids). The main challenges in terms of feasibility for the different types of species are breeding frequency and clutch sizes. Other differences (e.g., survival rates or efficiency of transgenesis or genome editing) have not been yet assayed.

### Developmental biology

To characterize gene-expression patterns and protein localization, several in situ hybridization (Fig. 8D) and antibody-staining protocols (Fig. 8F) have been optimized for cichlids. These include both chromogenic and fluorescent labeling techniques applied in embryos, adult tissues, and on sections [8, 11, 20, 23, 69, 203]. More recently, in situ hybridization chain reactions (HCR) that can fluorescently label transcripts of up to five genes has also been applied to study cichlid embryogenesis (Fig. 8E) [204]. Further, cartilage and skeletal stainings (Fig. 8G) were developed for several cichlid species to particularly address intra- and inter-specific variation in craniofacial, vertebral, and fin development [11, 22]. Finally, studying the ontogeny of cichlids throughout their complete development and adulthood and at cellular resolution (Fig. 8I) using repeated anesthesia and the use of epinephrine to contract pigment cells (to improve visualization of underlying tissues and ease quantifications) have allowed researchers to document embryo and juvenile pigmentation development [18].

### Morphological and behavioral phenotyping

Micro-computed tomography (μCT) scans have been repeatedly applied to measure variation in adult head and body shapes and other internal morphological features (e.g., pharyngeal bones; Fig. 8H) [6, 68, 108]. Further, 2D and 3D geometric morphometric analyses using a variety of software can be applied to characterize axes of morphological variation [6, 22, 68, 196]. Cichlids are also amenable to behavioral experiments in the field and in the lab to study collective behavior, social structures, kin selection, sensory systems, and neuroethology, among other topics [31, 133, 170].

## Research community and resources

The cichlid community has held bi-yearly Cichlid Science meetings since 2010, which have helped community engagement and growth. Several comprehensive reviews and books regarding the natural history, behavior, ecology, and evolution of these fishes have been published [33–35]. These, together with the recent advances in developmental genetics tools, allow for increasingly integrative and detailed studies.

There are several established inbred strains of cichlid species that have been in captivity for decades, such as certain populations of *A. burtoni* and *N. brichardi* [2]*.* Moreover, there are amelanistic lines that have been generated using CRISPR–Cas9 [29, 199] or derived from spontaneous mutations [205]. Importantly, most cichlid species can be kept in the laboratory, and most are easily collected in the field with the appropriate export permits. There are also multiple preserved cichlid collections spread throughout the world, some of which contain specimens that underwent whole-genome sequencing (e.g., University of Basel and University of Cambridge) [3, 6]. These represent unique collections that can be studied in future genotype–phenotype mapping studies.

Reference genomes of seven species representative of East African cichlid diversity have been sequenced, together with one South American cichlid outgroup (*A. citrinellus*) (Table 2). Furthermore, there are genome resequencing data for hundreds of species that can be used as a resource for speciation and adaptation genomics, character state and trait evolution reconstructions and phylogenomics projects (for example see [3, 4, 6]). Finally, there are also hundreds of RNAseq datasets for a variety of adult and embryonic tissues spanning the three major lakes in NCBI’s database.

**Table 2:**
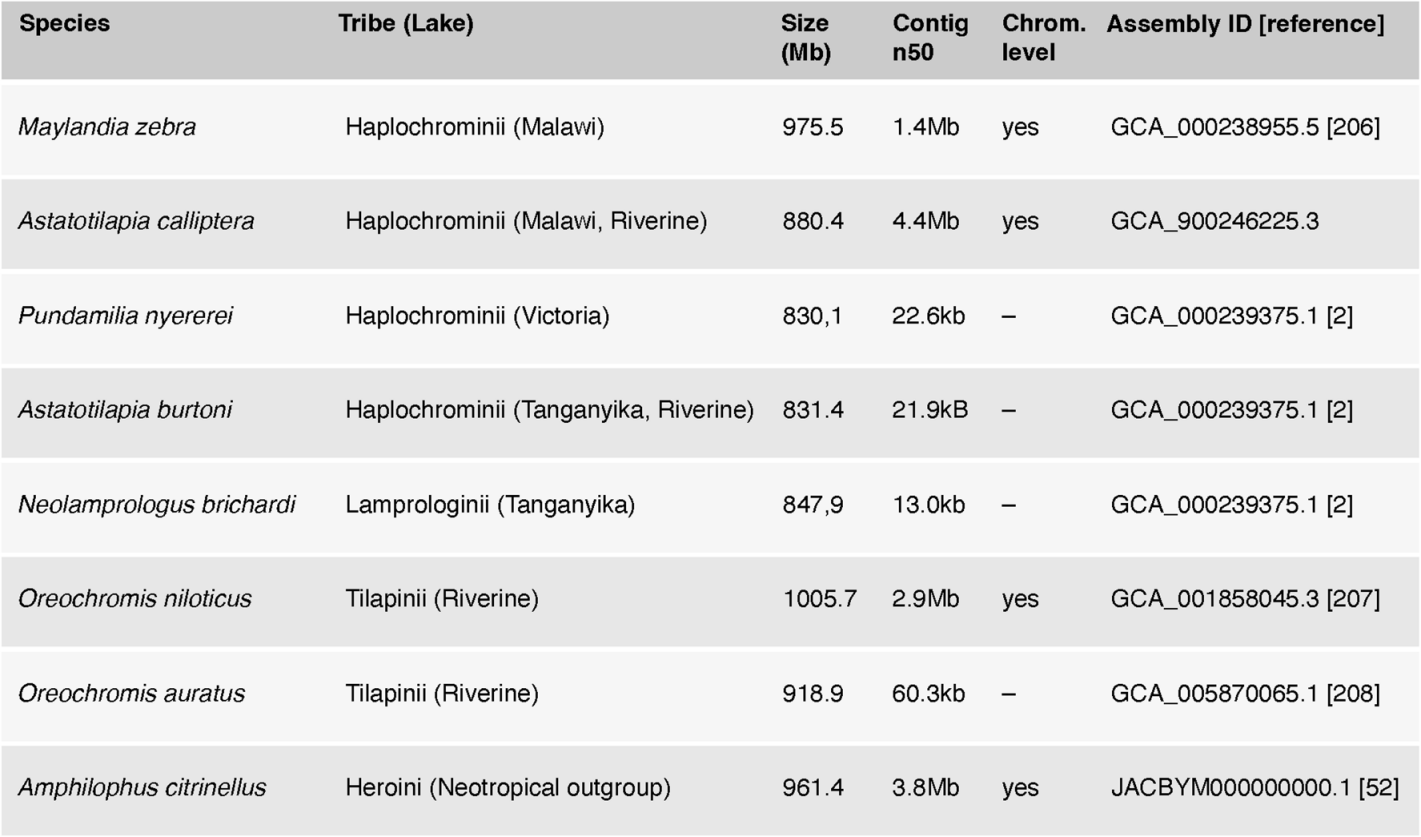
Overview of all cichlid reference genomes

The list includes all chromosome level (CL) genomes published or made accessible before 2022 and genomes available at Ensembl (release 106; April 2022)

## Data Availability

Not applicable.

## References

[1] Ring U, Albrecht C, Schrenk F, Hoorn MC, Perrigo A, Antonelli A (2018). The east African rift system tectonics, climate and biodiversity. Mt Clim Biodivers.

[2] Brawand D, Wagner CE, Li YI, Malinsky M, Keller I, Fan S (2014). The genomic substrate for adaptive radiation in African cichlid fish. Nature.

[3] Malinsky M, Svardal H, Tyers AM, Miska EA, Genner MJ, Turner GF (2018). Whole-genome sequences of Malawi cichlids reveal multiple radiations interconnected by gene flow. Nat Ecol Evol.

[4] McGee MD, Borstein SR, Meier JI, Marques DA, Mwaiko S, Taabu A (2020). The ecological and genomic basis of explosive adaptive radiation. Nature.

[5] Johnson TC, Scholz CA, Talbot MR, Kelts K, Ricketts RD, Ngobi G (1996). Late Pleistocene desiccation of Lake Victoria and rapid evolution of cichlid fishes. Science.

[6] Ronco F, Matschiner M, Böhne A, Boila A, Büscher HH, El Taher A (2021). Drivers and dynamics of a massive adaptive radiation in cichlid fishes. Nature.

[7] Wickler W (1962). ‘Egg-dummies’ as natural releasers in mouth-breeding cichlids. Nature.

[8] Santos ME, Braasch I, Boileau N, Meyer BS, Sauteur L, Böhne A (2014). The evolution of cichlid fish egg-spots is linked with a cis-regulatory change. Nat Commun.

[9] Sefc KM (2011). Mating and Parental Care in Lake Tanganyika’s Cichlids. Int J Evol Biol.

[10] Fujimura K, Okada N (2007). Development of the embryo, larva and early juvenile of Nile tilapia Oreochromis niloticus (Pisces: Cichlidae). Dev Growth Differ.

[11] Woltering JM, Holzem M, Schneider RF, Nanos V, Meyer A (2018). The skeletal ontogeny of Astatotilapia burtoni—a direct-developing model system for the evolution and development of the teleost body plan. BMC Dev Biol.

[12] Balon EK (1977). Early ontogeny of Labeotropbeus Ahl, 1927 (Mbuna, Cichlidae, Lake Malawi), with a discussion on advanced protective styles in fish reproduction and development. Environ Biol Fishes.

[13] Prusińska M, Mamcarz A, Kupren K (2009). Early ontogeny of Tropheus moorii Boulenger 1898 (Pisces, Cichlidae, Lake Tanganyika) in laboratory conditions. Pol J Nat Sci.

[14] Saemi-Komsari M, Mousavi-Sabet H, Kratochwil CF, Sattari M, Eagderi S, Meyer A (2018). Early developmental and allometric patterns in the electric yellow cichlid Labidochromis caeruleus. J Fish Biol.

[15] de Jong IML, Witte F, Richardson MK (2009). Developmental stages until hatching of the Lake Victoria cichlid Haplochromis piceatus (Teleostei: Cichlidae). J Morphol.

[16] Marconi A, Yang C, McKay S, Santos ME (2022). Morphological and temporal variation in early embryogenesis contributes to species divergence in Malawi cichlid fishes. bioRxiv.

[17] Heule C, Salzburger W (2011). The ontogenetic development of egg-spots in the haplochromine cichlid fish Astatotilapia burtoni. J Fish Biol.

[18] Liang Y, Gerwin J, Meyer A, Kratochwil CF (2020). Developmental and cellular basis of vertical bar color patterns in the East African Cichlid Fish Haplochromis latifasciatus. Front Cell Dev Biol.

[19] Hendrick LA, Carter GA, Hilbrands EH, Heubel BP, Schilling TF, Le Pabic P (2019). Bar, stripe and spot development in sand-dwelling cichlids from Lake Malawi. EvoDevo.

[20] Höch R, Schneider RF, Kickuth A, Meyer A, Woltering JM (2021). Spiny and soft-rayed fin domains in acanthomorph fish are established through a BMP-gremlin-shh signaling network. Proc Natl Acad Sci.

[21] le Pabic P, Stellwag EJ, Scemama J-L (2009). Embryonic development and Skeletogenesis of the Pharyngeal jaw apparatus in the Cichlid Nile Tilapia (Oreochromis niloticus). Anat Rec.

[22] Powder KE, Milch K, Asselin G, Albertson RC (2015). Constraint and diversification of developmental trajectories in cichlid facial morphologies. EvoDevo.

[23] Bloomquist RF, Fowler TE, Sylvester JB, Miro RJ, Streelman JT (2017). A compendium of developmental gene expression in Lake Malawi cichlid fishes. BMC Dev Biol.

[24] Kratochwil CF, Sefton MM, Meyer A (2015). Embryonic and larval development in the Midas cichlid fish species flock (*Amphilophus* spp.) a new evo-devo model for the investigation of adaptive novelties and species differences. BMC Dev Biol.

[25] Kimmel CB, Ballard WW, Kimmel SR, Ullmann B, Schilling TF (1995). Stages of embryonic development of the zebrafish. Dev Dyn.

[26] Kolm N, Goodwin NB, Balshine S, Reynolds JD (2006). Life history evolution in cichlids 1: revisiting the evolution of life histories in relation to parental care. J Evol Biol.

[27] Duponchelle F, Paradis E, Ribbink AJ, Turner GF (2008). Parallel life history evolution in mouthbrooding cichlids from the African Great Lakes. Proc Natl Acad Sci.

[28] Parsons PJ, Bridle JR, Rüber L, Genner MJ (2017). Evolutionary divergence in life history traits among populations of the Lake Malawi cichlid fish Astatotilapia calliptera. Ecol Evol.

[29] Li C-Y, Steighner JR, Sweatt G, Thiele TR, Juntti SA (2021). Manipulation of the Tyrosinase gene permits improved CRISPR/Cas editing and neural imaging in cichlid fish. Sci Rep.

[30] Kratochwil CF, Sefton MM, Liang Y, Meyer A (2017). Tol2 transposon-mediated transgenesis in the Midas cichlid (Amphilophus citrinellus)—towards understanding gene function and regulatory evolution in an ecological model system for rapid phenotypic diversification. BMC Dev Biol.

[31] Jordan A, Taborsky B, Taborsky M, Abate ME, Noakes DLG (2021). Cichlids as a Model System for Studying Social Behaviour and Evolution. Behav Ecol Evol Cichlid Fishes.

[32] Indermaur A, Theis A, Egger B, Salzburger W (2018). Mouth dimorphism in scale-eating cichlid fish from Lake Tanganyika advances individual fitness. Evolution.

[33] Abate ME, Noakes DL, Noakes DLG, Abate ME (2021). The Behavior, Ecology and Evolution of Cichlid Fishes. Parental Care in Cichlid Fishes.

[34] Barlow G (2000). The cichlid fishes: nature’s grand experiment in evolution.

[35] Fryer G, Iles TD. The cichlid fishes of the great lakes of Africa. Edinburgh: Oliver and Boyd; 1972

[36] Meyer A, Kocher TD, Basasibwaki P, Wilson AC (1990). Monophyletic origin of Lake Victoria cichlid fishes suggested by mitochondrial DNA sequences. Nature.

[37] Sturmbauer C, Meyer A (1992). Genetic divergence, speciation and morphological stasis in a lineage of African cichlid fishes. Nature.

[38] Joyce DA, Lunt DH, Genner MJ, Turner GF, Bills R, Seehausen O (2011). Repeated colonization and hybridization in Lake Malawi cichlids. Curr Biol.

[39] Mayer WE, Tichy H, Klein J (1998). Phylogeny of African cichlid fishes as revealed by molecular markers. Heredity.

[40] Erik V, Walter S, Jos S, Axel M (2003). Origin of the Superflock of Cichlid Fishes from Lake Victoria. East Africa Sci.

[41] Albertson RC, Markert JA, Danley PD, Kocher TD (1999). Phylogeny of a rapidly evolving clade: the cichlid fishes of Lake Malawi. East Africa Proc Natl Acad Sci.

[42] Wagner CE, Keller I, Wittwer S, Selz OM, Mwaiko S, Greuter L (2013). Genome-wide RAD sequence data provide unprecedented resolution of species boundaries and relationships in the Lake Victoria cichlid adaptive radiation. Mol Ecol.

[43] Takahashi T, Sota T (2016). A robust phylogeny among major lineages of the East African cichlids. Mol Phylogenet Evol.

[44] Irisarri I, Singh P, Koblmüller S, Torres-Dowdall J, Henning F, Franchini P (2018). Phylogenomics uncovers early hybridization and adaptive loci shaping the radiation of Lake Tanganyika cichlid fishes. Nat Commun.

[45] Meier JI, Marques DA, Mwaiko S, Wagner CE, Excoffier L, Seehausen O (2017). Ancient hybridization fuels rapid cichlid fish adaptive radiations. Nat Commun.

[46] Svardal H, Salzburger W, Malinsky M (2021). Genetic variation and hybridization in evolutionary radiations of Cichlid Fishes. Annu Rev Anim Biosci.

[47] Marques DA, Meier JI, Seehausen O (2019). A combinatorial view on speciation and adaptive radiation. Trends Ecol Evol.

[48] Gante HF, Matschiner M, Malmstrøm M, Jakobsen KS, Jentoft S, Salzburger W (2016). Genomics of speciation and introgression in Princess cichlid fishes from Lake Tanganyika. Mol Ecol.

[49] Svardal H, Quah FX, Malinsky M, Ngatunga BP, Miska EA, Salzburger W (2020). Ancestral hybridization facilitated species diversification in the lake Malawi Cichlid fish adaptive radiation. Mol Biol Evol.

[50] Elmer KR, Reggio C, Wirth T, Verheyen E, Salzburger W, Meyer A (2009). Pleistocene desiccation in East Africa bottlenecked but did not extirpate the adaptive radiation of Lake Victoria haplochromine cichlid fishes. Proc Natl Acad Sci.

[51] Malinsky M, Challis RJ, Tyers AM, Schiffels S, Terai Y, Ngatunga BP (2015). Genomic islands of speciation separate cichlid ecomorphs in an East African crater lake. Science.

[52] Kautt AF, Kratochwil CF, Nater A, Machado-Schiaffino G, Olave M, Henning F (2020). Contrasting signatures of genomic divergence during sympatric speciation. Nature.

[53] Schluter D (2000). The ecology of adaptive radiation.

[54] Wagner CE, Harmon LJ, Seehausen O (2012). Ecological opportunity and sexual selection together predict adaptive radiation. Nature.

[55] Schneider RF, Meyer A (2017). How plasticity, genetic assimilation and cryptic genetic variation may contribute to adaptive radiations. Mol Ecol.

[56] Hulsey CD (2006). Function of a key morphological innovation: fusion of the cichlid pharyngeal jaw. Proc R Soc B Biol Sci.

[57] Carleton KL, Conte MA, Malinsky M, Nandamuri SP, Sandkam BA, Meier JI (2020). Movement of transposable elements contributes to cichlid diversity. Mol Ecol.

[58] Salzburger W (2018). Understanding explosive diversification through cichlid fish genomics. Nat Rev Genet.

[59] Kratochwil CF, Meyer A (2015). Closing the genotype–phenotype gap: Emerging technologies for evolutionary genetics in ecological model vertebrate systems. BioEssays.

[60] Seehausen O, Terai Y, Magalhaes IS, Carleton KL, Mrosso HDJ, Miyagi R (2008). Speciation through sensory drive in cichlid fish. Nature.

[61] Rometsch SJ, Torres-Dowdall J, Meyer A (2020). Evolutionary dynamics of pre- and postzygotic reproductive isolation in cichlid fishes. Philos Trans R Soc B Biol Sci.

[62] Arendt J, Reznick D (2008). Convergence and parallelism reconsidered: what have we learned about the genetics of adaptation?. Trends Ecol Evol.

[63] Barghi N, Hermisson J, Schlötterer C (2020). Polygenic adaptation: a unifying framework to understand positive selection. Nat Rev Genet.

[64] Fagny M, Austerlitz F (2021). Polygenic Adaptation: Integrating Population Genetics and Gene Regulatory Networks. Trends Genet.

[65] Stern DL (2013). The genetic causes of convergent evolution. Nat Rev Genet.

[66] Elmer KR, Meyer A (2011). Adaptation in the age of ecological genomics: insights from parallelism and convergence. Trends Ecol Evol.

[67] Albertson RC, Kocher TD (2006). Genetic and developmental basis of cichlid trophic diversity. Heredity.

[68] Muschick M, Indermaur A, Salzburger W (2012). Convergent Evolution within an Adaptive Radiation of Cichlid Fishes. Curr Biol.

[69] Kratochwil CF, Yipeng L, Jan G, Woltering JM, Sabine U, Frederico H (2018). Agouti-related peptide 2 facilitates convergent evolution of stripe patterns across cichlid fish radiations. Science.

[70] Pardo-Diaz C, Salazar C, Jiggins CD (2015). Towards the identification of the loci of adaptive evolution. Methods Ecol Evol.

[71] Kratochwil CF, Meyer A (2015). Evolution: tinkering within gene regulatory landscapes. Curr Biol.

[72] Mehta TK, Penso-Dolfin L, Nash W, Roy S, Di-Palma F, Haerty W (2021). Evolution of miRNA binding sites and regulatory networks in cichlids. J bioRxiv.

[73] Franchini P, Xiong P, Fruciano C, Schneider RF, Woltering JM, Hulsey CD (2019). MicroRNA Gene Regulation in Extremely Young and Parallel Adaptive Radiations of Crater Lake Cichlid Fish. Mol Biol Evol.

[74] Loh Y-HE, Yi SV, Streelman JT (2011). Evolution of MicroRNAs and the diversification of species. Genome Biol Evol.

[75] Vernaz G, Malinsky M, Svardal H, Du M, Tyers AM, Santos ME (2021). Mapping epigenetic divergence in the massive radiation of Lake Malawi cichlid fishes. Nat Commun.

[76] Kratochwil CF, Meyer A (2015). Mapping active promoters by ChIP-seq profiling of H3K4me3 in cichlid fish—a first step to uncover cis-regulatory elements in ecological model teleosts. Mol Ecol Resour.

[77] Kratochwil CF, Liang Y, Urban S, Torres-Dowdall J, Meyer A (2019). Evolutionary dynamics of structural variation at a key locus for color pattern diversification in Cichlid Fishes. Genome Biol Evol.

[78] Fan S, Meyer A (2014). Evolution of genomic structural variation and genomic architecture in the adaptive radiations of African cichlid fishes. Front Genet.

[79] Conte MA, Joshi R, Moore EC, Nandamuri SP, Gammerdinger WJ, Roberts RB (2019). Chromosome-scale assemblies reveal the structural evolution of African cichlid genomes. GigaScience.

[80] Alphen JV (1994). Evolution of colour patterns in East African cichlid fish. J Evol Biol.

[81] Urban S, Gerwin J, Hulsey CD, Meyer A, Kratochwil CF (2022). The repeated evolution of stripe patterns is correlated with body morphology in the adaptive radiations of East African cichlid fishes. Ecol Evol.

[82] Liang Y, Grauvogl M, Meyer A, Kratochwil CF (2021). Functional conservation and divergence of color-pattern-related agouti family genes in teleost fishes. J Exp Zoolog B Mol Dev Evol.

[83] Gerwin J, Urban S, Meyer A, Kratochwil CF (2021). Of bars and stripes: A Malawi cichlid hybrid cross provides insights into genetic modularity and evolution of modifier loci underlying colour pattern diversification. Mol Ecol.

[84] Urban S, Nater A, Meyer A, Kratochwil CF (2021). Different Sources of Allelic Variation Drove Repeated Color Pattern Divergence in Cichlid Fishes. Mol Biol Evol.

[85] Kratochwil CF, Liang Y, Gerwin J, Franchini P, Meyer A (2022). Comparative ontogenetic and transcriptomic analyses shed light on color pattern divergence in cichlid fishes. Evol Dev.

[86] Henning F, Meyer A (2012). Eggspot Number and Sexual Selection in the Cichlid Fish *Astatotilapia burtoni*. PLoS ONE.

[87] Theis A, Salzburger W, Egger B (2012). The function of anal fin egg-spots in the Cichlid Fish *Astatotilapia burtoni*. PLoS ONE.

[88] Theis A, Roth O, Cortesi F, Ronco F, Salzburger W, Egger B (2017). Variation of anal fin egg-spots along an environmental gradient in a haplochromine cichlid fish. Evolution.

[89] Salzburger W, Braasch I, Meyer A (2007). Adaptive sequence evolution in a color gene involved in the formation of the characteristic egg-dummies of male haplochromine cichlid fishes. BMC Biol.

[90] Santos ME, Baldo L, Gu L, Boileau N, Musilova Z, Salzburger W (2016). Comparative transcriptomics of anal fin pigmentation patterns in cichlid fishes. BMC Genomics.

[91] Roberts RB, Ser JR, Kocher TD (2009). sexual conflict resolved by invasion of a novel sex determiner in lake Malawi Cichlid Fishes. Science.

[92] Streelman JT, Albertson RC, Kocher TD (2003). Genome mapping of the orange blotch colour pattern in cichlid fishes. Mol Ecol.

[93] Parnell NF, Streelman JT (2013). Genetic interactions controlling sex and color establish the potential for sexual conflict in Lake Malawi cichlid fishes. Heredity.

[94] Albertson RC, Powder KE, Hu Y, Coyle KP, Roberts RB, Parsons KJ (2014). Genetic basis of continuous variation in the levels and modular inheritance of pigmentation in cichlid fishes. Mol Ecol.

[95] Feller AF, Haesler MP, Peichel CL, Seehausen O (2020). Genetic architecture of a key reproductive isolation trait differs between sympatric and non-sympatric sister species of Lake Victoria cichlids. Proc R Soc B Biol Sci.

[96] Ahi EP, Lecaudey LA, Ziegelbecker A, Steiner O, Goessler W, Sefc KM (2020). Expression levels of the tetratricopeptide repeat protein gene *ttc39b* covary with carotenoid-based skin colour in cichlid fish. Biol Lett.

[97] Ahi EP, Lecaudey LA, Ziegelbecker A, Steiner O, Glabonjat R, Goessler W (2020). Comparative transcriptomics reveals candidate carotenoid color genes in an East African cichlid fish. BMC Genomics.

[98] Border SE, Piefke TJ, Fialkowski RJ, Tryc MR, Funnell TR, DeOliveira GM (2019). Color change and pigmentation in a color polymorphic cichlid fish. Hydrobiologia.

[99] Liang Y, Meyer A, Kratochwil CF (2020). Neural innervation as a potential trigger of morphological color change and sexual dimorphism in cichlid fish. Sci Rep.

[100] Alvarado SG (2020). Molecular plasticity in animal pigmentation: emerging processes underlying color changes. Integr Comp Biol.

[101] Muske LE, Fernald RD (1987). Control of a teleost social signal. J Comp Physiol A.

[102] Powder KE, Albertson RC (2016). Cichlid fishes as a model to understand normal and clinical craniofacial variation. Dev Biol.

[103] Ronco F, Salzburger W (2021). Tracing evolutionary decoupling of oral and pharyngeal jaws in cichlid fishes. Evol Lett.

[104] Craig AR, Streelman J (2003). Todd, Kocher Thomas D. Directional selection has shaped the oral jaws of Lake Malawi cichlid fishes. Proc Natl Acad Sci.

[105] Powder KE, Cousin H, McLinden GP, Craig AR (2014). A nonsynonymous mutation in the transcriptional regulator lbh is associated with Cichlid craniofacial adaptation and neural crest cell development. Mol Biol Evol.

[106] Roberts RB, Hu Y, Albertson RC, Kocher TD (2011). Craniofacial divergence and ongoing adaptation via the hedgehog pathway. Proc Natl Acad Sci.

[107] Albertson RC, Streelman JT, Kocher TD, Yelick PC (2005). Integration and evolution of the cichlid mandible: The molecular basis of alternate feeding strategies. Proc Natl Acad Sci.

[108] Conith AJ, Albertson RC (2021). The cichlid oral and pharyngeal jaws are evolutionarily and genetically coupled. Nat Commun.

[109] Bloomquist RF, Parnell NF, Phillips KA, Fowler TE, Yu TY, Sharpe PT (2015). Coevolutionary patterning of teeth and taste buds. Proc Natl Acad Sci.

[110] Hulsey CD, Machado-Schiaffino G, Keicher L, Ellis-Soto D, Henning F, Meyer A (2017). The integrated genomic architecture and evolution of dental divergence in East African Cichlid fishes (Haplochromis chilotes x H. nyererei). G3.

[111] Fraser GJ, Hulsey CD, Bloomquist RF, Uyesugi K, Manley NR, Streelman JT (2009). An ancient gene network is co-opted for teeth on old and new jaws. PLOS Biol.

[112] RÜber L, Adams DC (2001). Evolutionary convergence of body shape and trophic morphology in cichlids from Lake Tanganyika. J Evol Biol.

[113] Hulsey CD, Roberts RJ, Loh Y-HE, Rupp MF, Streelman JT. Lake Malawi cichlid evolution along a benthic/limnetic axis. Ecol Evol. 2013;3:2262–72. 10.1002/ece3.633 PMC3728963 23919168

[114] Kocher TD (2004). Adaptive evolution and explosive speciation: the cichlid fish model. Nat Rev Genet.

[115] DeLorenzo L, Mathews D, Brandon AA, Joglekar M, Baez AC, Moore EC, et al. Genetic basis of body shape variation along the benthic-pelagic axis in cichlid fishes. bioRxiv. 2021;2021.10.02.462884.

[116] Ahi EP, Richter F, Sefc KM (2017). A gene expression study of ornamental fin shape in Neolamprologus brichardi, an African cichlid species. Sci Rep.

[117] Ahi EP, Richter F, Lecaudey LA, Sefc KM (2019). Gene expression profiling suggests differences in molecular mechanisms of fin elongation between cichlid species. Sci Rep.

[118] Navon D, Olearczyk N, Albertson RC (2017). Genetic and developmental basis for fin shape variation in African cichlid fishes. Mol Ecol.

[119] Albertson RC, Kawasaki KC, Tetrault ER, Powder KE (2018). Genetic analyses in Lake Malawi cichlids identify new roles for Fgf signaling in scale shape variation. Commun Biol.

[120] Wagner M, Bračun S, Duenser A, Sturmbauer C, Gessl W, Ahi EP (2022). Expression variations in ectodysplasin-A gene (eda) may contribute to morphological divergence of scales in haplochromine cichlids. BMC Ecol Evol.

[121] Escobar-Camacho D, Carleton KL (2015). Sensory modalities in cichlid fish behavior. Curr Opin Behav Sci.

[122] Carleton KL, Yourick MR (2020). Axes of visual adaptation in the ecologically diverse family Cichlidae. Semin Cell Dev Biol.

[123] Ricci V, Ronco F, Musilova Z, Salzburger W (2022). Molecular evolution and depth-related adaptations of rhodopsin in the adaptive radiation of cichlid fishes in Lake Tanganyika. Mol Ecol.

[124] Sugawara T, Terai Y, Imai H, Turner GF, Koblmüller S, Sturmbauer Christian, et al. Parallelism of amino acid changes at the RH1 affecting spectral sensitivity among deep-water cichlids from Lakes Tanganyika and Malawi. Proc Natl Acad Sci. 2005;102:5448–53. 10.1073/pnas.0405302102 PMC556224 15809435

[125] Spady TC, Seehausen O, Loew ER, Jordan RC, Kocher TD, Carleton KL (2005). Adaptive Molecular Evolution in the Opsin Genes of Rapidly Speciating Cichlid Species. Mol Biol Evol.

[126] O’Quin KE, Smith D, Naseer Z, Schulte J, Engel SD, Loh Y-HE, et al. Divergence in cis-regulatory sequences surrounding the opsin gene arrays of African cichlid fishes. BMC Evol Biol. 2011;11:120. 10.1186/1471-2148-11-120 PMC3116502 21554730

[127] Thünken T, Bakker TCM, Baldauf SA (2014). “Armpit effect” in an African cichlid fish: self-referent kin recognition in mating decisions of male Pelvicachromis taeniatus. Behav Ecol Sociobiol.

[128] Keller-Costa T, Canário AVM, Hubbard PC (2015). Chemical communication in cichlids: A mini-review. Gen Comp Endocrinol.

[129] Blais J, Plenderleith M, Rico C, Taylor MI, Seehausen O, Van Oosterhout C, et al. Assortative mating among Lake Malawi cichlid fish populations is not simply predictable from male nuptial colour *blais J. BMC Evol Biol. 2009;9. 10.1186/1471-2148-9-53 PMC2667177 19265521

[130] Plenderleith M, van Oosterhout C, Robinson RL, Turner GF (2005). Female preference for conspecific males based on olfactory cues in a Lake Malawi cichlid fish. Biol Lett.

[131] Hubbard PC, Mota VC, Keller-Costa T, da Silva JP, Canário AVM. Chemical communication in tilapia: A comparison of Oreochromis mossambicus with O. niloticus. Gen Comp Endocrinol. 2014;207:13–20. 10.1016/j.ygcen.2014.06.022 24979336

[132] Miranda A, Almeida OG, Hubbard PC, Barata EN, Canário AVM (2005). Olfactory discrimination of female reproductive status by male tilapia(Oreochromis mossambicus). J Exp Biol.

[133] Nikonov AA, Maruska KP (2019). Male dominance status regulates odor-evoked processing in the forebrain of a cichlid fish. Sci Rep.

[134] Verzijden MN, ten Cate C (2007). Early learning influences species assortative mating preferences in Lake Victoria cichlid fish. Biol Lett Royal Society.

[135] Verzijden MN, Korthof REM, ten Cate C. Females learn from mothers and males learn from others. The effect of mother and siblings on the development of female mate preferences and male aggression biases in Lake Victoria cichlids, genus Mbipia. Behav Ecol Sociobiol. 2008;62:1359–68.

[136] Hansen A, Zielinski BS (2005). Diversity in the olfactory epithelium of bony fishes: Development, lamellar arrangement, sensory neuron cell types and transduction components. J Neurocytol.

[137] Azzouzi N, Barloy-Hubler F, Galibert F. Inventory of the cichlid olfactory receptor gene repertoires: Identification of olfactory genes with more than one coding exon. BMC Genomics. 2014;15. 10.1186/1471-2164-15-586 PMC4122780 25015101

[138] Nikaido M, Suzuki H, Toyoda A, Fujiyama A, Hagino-Yamagishi K, Kocher TD (2013). Lineage-Specific Expansion of Vomeronasal Type 2 Receptor-Like (OlfC) Genes in Cichlids May Contribute to Diversification of Amino Acid Detection Systems. Genome Biol Evol.

[139] Ota T, Nikaido M, Suzuki H, Hagino-Yamagishi K, Okada N (2012). Characterization of V1R receptor (ora) genes in Lake Victoria cichlids. Gene.

[140] Nikaido M, Ota T, Hirata T, Suzuki H, Satta Y, Aibara M (2014). Multiple Episodic Evolution Events in V1R Receptor Genes of East-African Cichlids. Genome Biol Evol.

[141] Bertucci F, Attia J, Beauchaud M, Mathevon N (2012). Sounds produced by the cichlid fish Metriaclima zebra allow reliable estimation of size and provide information on individual identity. J Fish Biol.

[142] Longrie N, Poncin P, Denoël M, Gennotte V, Delcourt J, Parmentier E (2013). Behaviours Associated with Acoustic Communication in Nile Tilapia (Oreochromis niloticus). PLoS ONE.

[143] Maruska KP, Ung US, Fernald RD (2012). The African Cichlid Fish Astatotilapia burtoni Uses Acoustic Communication for Reproduction: Sound Production, Hearing, and Behavioral Significance. PLoS ONE.

[144] Danley PD, Husemann M, Chetta J (2012). Acoustic diversity in Lake Malawi’s rock-dwelling cichlids. Environ Biol Fishes.

[145] Amorim MCP, Simões JM, Fonseca PJ, Turner GF. Species differences in courtship acoustic signals among five Lake Malawi cichlid species (Pseudotropheus spp.). J Fish Biol. 2008;72:1355–68.

[146] Popper AN, Fay RR (2011). Rethinking sound detection by fishes. Hear Res.

[147] Schulz-Mirbach T, Heß M, Metscher BD, Ladich F (2013). A unique swim bladder-inner ear connection in a teleost fish revealed by a combined high-resolution microtomographic and three-dimensional histological study. BMC Biol.

[148] Lobel PS, Garner JG, Kaatz IM, Rice AN, Abate ME, Noakes DLG (2021). Sonic Cichlids. Behav Ecol Evol Cichlid Fishes.

[149] Webb JF. Morphological diversity, development, and evolution of the mechanosensory lateral line system. Lateral Line Syst. 2014;17–72.

[150] Webb JF (1989). Gross Morphology and Evolution of the Mechanoreceptive Lateral-Line System in Teleost Fishes (Part 1 of 2). Brain Behav Evol.

[151] Webb JF, Maruska KP, Butler JM, Schwalbe MAB, Abate ME, Noakes DLG (2021). The Mechanosensory Lateral Line System of Cichlid Fishes: From Anatomy to Behavior. Behav Ecol Evol Cichlid Fishes.

[152] Edgley DE, Genner MJ. Adaptive Diversification of the Lateral Line System during Cichlid Fish Radiation. iScience. 2019;16:1–11. 10.1016/j.isci.2019.05.016 PMC6542376 31146127

[153] Schwalbe MAB, Bassett DK, Webb JF (2012). Feeding in the dark: lateral-line-mediated prey detection in the peacock cichlid Aulonocara stuartgranti. J Exp Biol.

[154] Bird NC, Webb JF (2014). Heterochrony, modularity, and the functional evolution of the mechanosensory lateral line canal system of fishes. EvoDevo.

[155] Webb JF, Bird NC, Carter L, Dickson J (2014). Comparative development and evolution of two lateral line phenotypes in Lake Malawi cichlids. J Morphol.

[156] Gammerdinger WJ, Kocher TD. Unusual Diversity of Sex Chromosomes in African Cichlid Fishes. Genes. 2018;9. 10.3390/genes9100480 PMC6210639 30287777

[157] El Taher Athimed, Ronco Fabrizia, Matschiner Michael, Salzburger Walter, Böhne Astrid. Dynamics of sex chromosome evolution in a rapid radiation of cichlid fishes. Sci Adv. 7:eabe8215. 10.1126/sciadv.abe8215 PMC8442896 34516923

[158] Feller AF, Ogi V, Seehausen O, Meier JI (2021). Identification of a novel sex determining chromosome in cichlid fishes that acts as XY or ZW in different lineages. Hydrobiologia.

[159] Ser JR, Roberts RB, Kocher TD (2010). Multiple Interacting Loci Control Sex Determination in Lake Malawi Cichlid Fish. Evolution.

[160] Roberts NB, Juntti SA, Coyle KP, Dumont BL, Stanley MK, Ryan AQ (2016). Polygenic sex determination in the cichlid fish Astatotilapia burtoni. BMC Genomics.

[161] Munby H, Linderoth T, Fischer B, Du M, Vernaz G, Tyers AM, et al. Differential use of multiple genetic sex determination systems in divergent ecomorphs of an African crater lake cichlid. bioRxiv. 2021;2021.08.05.455235.

[162] Moore EC, Ciccotto PJ, Peterson EN, Lamm MS, Albertson RC, Roberts RB (2022). Polygenic sex determination produces modular sex polymorphism in an African cichlid fish. Proc Natl Acad Sci.

[163] Roth G (2015). Convergent evolution of complex brains and high intelligence. Philos Trans R Soc B Biol Sci.

[164] Bshary R, Wickler W, Fricke H (2002). Fish cognition: a primate’s eye view. Anim Cogn.

[165] Hofmann HA, Benson ME, Fernald RD (1999). Social status regulates growth rate: Consequences for life-history strategies. Proc Natl Acad Sci.

[166] Grosenick L, Clement TS, Fernald RD (2007). Fish can infer social rank by observation alone. Nature.

[167] Maruska KP, Fernald RD (2010). Behavioral and physiological plasticity: Rapid changes during social ascent in an African cichlid fish. Horm Behav.

[168] Taborsky M, Dickinson JL, Koenig WD (2016). Cichlid fishes: A model for the integrative study of social behavior. Coop Breed Vertebr Stud Ecol Evol Behav.

[169] York RA, Patil C, Abdilleh K, Johnson, Conte MA, Genner MJ, et al. Behavior-dependent cis regulation reveals genes and pathways associated with bower building in cichlid fishes. Proc Natl Acad Sci. 2018;115:E11081–90. 10.1073/pnas.1810140115 PMC6255178 30397142

[170] Juntti SA, Hilliard AT, Kent KR, Kumar A, Nguyen A, Jimenez MA (2016). A Neural Basis for Control of Cichlid Female Reproductive Behavior by Prostaglandin F2α. Curr Biol.

[171] Alward BA, Laud VA, Skalnik CJ, York RA, Juntti SA, Fernald RD (2020). Modular genetic control of social status in a cichlid fish. Proc Natl Acad Sci.

[172] Sylvester JB, Rich CA, Loh Y-HE, van Staaden MJ, Fraser GJ, Streelman JT. Brain diversity evolves via differences in patterning. Proc Natl Acad Sci. 2010;107:9718–23. 10.1073/pnas.1000395107 PMC2906875 20439726

[173] Sylvester JB, Rich CA, Yi C, Peres JN, Houart C, Streelman JT (2013). Competing signals drive telencephalon diversity. Nat Commun.

[174] West-Eberhard MJ (1989). Phenotypic Plasticity and the Origins of Diversity. Annu Rev Ecol Syst.

[175] Karagic N, Meyer A, Hulsey CD (2020). Phenotypic Plasticity in Vertebrate Dentitions. Integr Comp Biol.

[176] Schneider RF, Li Y, Meyer A, Gunter HM (2014). Regulatory gene networks that shape the development of adaptive phenotypic plasticity in a cichlid fish. Mol Ecol.

[177] Ghalambor CK, Hoke KL, Ruell EW, Fischer EK, Reznick DN, Hughes KA (2015). Non-adaptive plasticity potentiates rapid adaptive evolution of gene expression in nature. Nature.

[178] Baldo L, Riera JL, Tooming-Klunderud A, Albà MM, Salzburger W (2015). Gut Microbiota Dynamics during Dietary Shift in Eastern African Cichlid Fishes. PLoS ONE.

[179] Singh A, Faber-Hammond JJ, O’Rourke CF, Renn SCP (2019). Gut microbial diversity increases with social rank in the African cichlid fish. Astatotilapia burtoni Anim Behav.

[180] Seehausen O, van Alphen JJM, Witte F (1997). Cichlid Fish diversity threatened by eutrophication that curbs sexual selection. Science.

[181] McGee MD, Borstein SR, Neches RY, Buescher HH, Seehausen O, Wainwright PC (2015). A pharyngeal jaw evolutionary innovation facilitated extinction in Lake Victoria cichlids. Science.

[182] Martin CH, Cutler JS, Friel JP, Dening Touokong C, Coop G, Wainwright PC (2015). Complex histories of repeated gene flow in Cameroon crater lake cichlids cast doubt on one of the clearest examples of sympatric speciation. Evolution.

[183] Musilova Z, Indermaur A, Bitja-Nyom AR, Omelchenko D, Kłodawska M, Albergati L (2019). Evolution of the visual sensory system in cichlid fishes from crater lake Barombi Mbo in Cameroon. Mol Ecol.

[184] Poelstra JW, Richards EJ, Martin CH (2018). Speciation in sympatry with ongoing secondary gene flow and a potential olfactory trigger in a radiation of Cameroon cichlids. Mol Ecol.

[185] Barluenga M, Stölting KN, Salzburger W, Muschick M, Meyer A (2006). Sympatric speciation in Nicaraguan crater lake cichlid fish. Nature.

[186] Römer U, Beisenherz W (1996). Environmental determination of sex in Apistogrammai (Cichlidae) and two other freshwater fishes (Teleostei). J Fish Biol.

[187] Matschiner M, Böhne A, Ronco F, Salzburger W (2020). The genomic timeline of cichlid fish diversification across continents. Nat Commun.

[188] Myles C, Wayne M (2008). Quantitative trait locus (QTL) analysis. Nat Educ.

[189] Bush WS, Moore JH (2012). Genome-wide association studies. PLOS Comput Biol.

[190] Kang HM, Sul JH, Service SK, Zaitlen NA, Kong S, Freimer NB, et al. 2010 Variance component model to account for sample structure in genome wide association studies. J Nat Genet. 10.1038/ng.548 10.1038/ng.548 PMC3092069 20208533

[191] El Taher A, Ronco F, Matschiner M, Salzburger W, Böhne A (2021). Dynamics of sex chromosome evolution in a rapid radiation of cichlid fishes. Sci Adv.

[192] Kratochwil CF, Kautt AF, Nater A, Härer A, Liang Y, Henning F (2022). An intronic transposon insertion associates with a trans-species color polymorphism in Midas cichlid fishes. Nat Commun.

[193] Roberts RB, Moore EC, Kocher TD (2017). An allelic series at pax7a is associated with colour polymorphism diversity in Lake Malawi cichlid fish. Mol Ecol.

[194] Hu Y, Albertson RC (2014). Hedgehog signaling mediates adaptive variation in a dynamic functional system in the cichlid feeding apparatus. Proc Natl Acad Sci.

[195] Fraser GJ, Bloomquist RF, Streelman JT (2013). Common developmental pathways link tooth shape to regeneration. Dev Biol.

[196] Parsons KJ, Trent Taylor A, Powder KE, Albertson RC (2014). Wnt signalling underlies the evolution of new phenotypes and craniofacial variability in Lake Malawi cichlids. Nat Commun.

[197] Li M, Yang H, Zhao J, Fang L, Shi H, Li M (2014). Efficient and Heritable Gene Targeting in Tilapia by CRISPR/Cas9. Genetics.

[198] Li M, Liu X, Dai S, Xiao H, Wang D (2019). High efficiency targeting of non-coding sequences using CRISPR/Cas9 system in Tilapia. G3.

[199] Clark B, Elkin J, Marconi A, Turner GF, Smith AM, Joyce D (2022). Oca2 targeting using CRISPR/Cas9 in the Malawi cichlid Astatotilapia calliptera. R Soc Open Sci.

[200] Fujimura K, Kocher TD (2011). Tol2-mediated transgenesis in tilapia (Oreochromis niloticus). Aquaculture.

[201] Juntti SA, Hu CK, Fernald RD (2013). Tol2-Mediated Generation of a Transgenic Haplochromine Cichlid. Astatotilapia burtoni PLOS ONE.

[202] Seleit A, Aulehla A, Paix A (2021). Endogenous protein tagging in medaka using a simplified CRISPR/Cas9 knock-in approach. Elife.

[203] Dalton BE, Lu J, Leips J, Cronin TW, Carleton KL (2015). Variable light environments induce plastic spectral tuning by regional opsin coexpression in the African cichlid fish. Metriaclima zebra Mol Ecol.

[204] Choi HMT, Schwarzkopf M, Fornace ME, Acharya A, Artavanis G, Stegmaier J (2018). Third-generation in situ hybridization chain reaction: multiplexed, quantitative, sensitive, versatile, robust. Development.

[205] Kratochwil CF, Urban S, Meyer A (2019). Genome of the Malawi golden cichlid fish (Melanochromis auratus) reveals exon loss of oca2 in an amelanistic morph. Pigment Cell Melanoma Res.

[206] Conte MA, Kocher TD (2015). An improved genome reference for the African cichlid. Metriaclima zebra BMC Genomics.

[207] Conte MA, Gammerdinger WJ, Bartie KL, Penman DJ, Kocher TD (2017). A high quality assembly of the Nile Tilapia (Oreochromis niloticus) genome reveals the structure of two sex determination regions. BMC Genomics.

[208] Bian C, Li J, Lin X, Chen X, Yi Y, You X (2019). Whole Genome sequencing of the blue tilapia (Oreochromis aureus) provides a valuable genetic resource for biomedical research on tilapias. Mar Drugs.

